# Epithelial cells detect functional type III secretion system of enteropathogenic *Escherichia coli* through a novel NF-κB signaling pathway

**DOI:** 10.1371/journal.ppat.1006472

**Published:** 2017-07-03

**Authors:** Yael Litvak, Shir Sharon, Meirav Hyams, Li Zhang, Simi Kobi, Naama Katsowich, Shira Dishon, Gabriel Nussbaum, Na Dong, Feng Shao, Ilan Rosenshine

**Affiliations:** 1 Department of Microbiology and Molecular Genetics, Institute of Medical Research Israel-Canada, Faculty of Medicine, The Hebrew University of Jerusalem, Jerusalem, Israel; 2 National Laboratory of Biomacromolecules, Institute of Biophysics, Chinese Academy of Sciences, Beijing, China; National Institute of Biological Sciences, Beijing, China; 3 The Institute of Dental Sciences, Hebrew University-Hadassah Faculty of Dental Medicine, Jerusalem, Israel; University of Michigan Medical School, UNITED STATES

## Abstract

Enteropathogenic *Escherichia coli* (EPEC), a common cause of infant diarrhea, is associated with high risk of mortality in developing countries. The primary niche of infecting EPEC is the apical surface of intestinal epithelial cells. EPEC employs a type three secretion system (TTSS) to inject the host cells with dozens of effector proteins, which facilitate attachment to these cells and successful colonization. Here we show that EPEC elicit strong NF-κB activation in infected host cells. Furthermore, the data indicate that active, pore-forming TTSS *per se* is necessary and sufficient for this NF-κB activation, regardless of any specific effector or protein translocation. Importantly, upon infection with wild type EPEC this NF-κB activation is antagonized by anti-NF-κB effectors, including NleB, NleC and NleE. Accordingly, this NF-κB activation is evident only in cells infected with EPEC mutants deleted of *nleB*, *nleC*, and *nleE*. The TTSS-dependent NF-κB activation involves a unique pathway, which is independent of TLRs and Nod1/2 and converges with other pathways at the level of TAK1 activation. Taken together, our results imply that epithelial cells have the capacity to sense the EPEC TTSS and activate NF-κB in response. Notably, EPEC antagonizes this capacity by delivering anti-NF-κB effectors into the infected cells.

## Author summary

The intestine harbors a dense community of commensal bacteria that play a vital role in host health and homeostasis, but it is also the port of entry for many pathogens. An important function of the intestinal epithelial cells is coordinating the immune response to microbial signals, ranging from tolerance towards beneficial species to a robust anti-pathogen immune response. The mechanisms underlying the ability of intestinal epithelial cells to specifically distinguish pathogens from commensal bacteria are only partially understood. Commensal and enteropathogenic *E*. *coli* strains are highly similar and both reside in the gut lumen. However, only the pathogen is equipped with a type III protein secretion system, which is employed to inject effector proteins into the host cells. Here we show that epithelial cells distinguish the pathogenic from the highly similar commensal *E*. *coli* strains through specific sensing of the TTSS activity and respond by triggering a novel defense signaling pathway, but the pathogen attempt to avoid its detection by injection effectors that block the defense signaling.

## Introduction

Enteropathogenic and enterohemorrhagic *E*. *coli* (EPEC and EHEC, respectively) are important human pathogens that cause symptoms ranging from subclinical chronic colonization to acute, life threatening infections [1]. EPEC and EHEC form typical attaching and effacing (AE) lesions on intestinal epithelial cells. These lesions are characterized by intimate attachment to the epithelium and effacement of the brush border microvilli [2,3]. Throughout infection, these pathogens remain either in the intestinal lumen, or attached to the apical surface of the intestinal epithelia. From this extracellular location these pathogens manipulate epithelial cell functions to facilitate efficient host colonization [2,4]. Although the apical surface of the intestinal epithelium is constantly challenged with massive amounts of MAMPs (Microbial Associated Molecular Patterns), the reaction to these MAMPs is tightly regulated and restrained to prevent chronic inflammation. MAMPs, such as LPS, flagellin and CpG DNA, derived from commensal or pathogenic *E*. *coli*, are identical. Yet, the epithelium cells must distinguish commensal from pathogenic bacteria in order to maintain tolerance towards the beneficial commensal bacteria, while unleashing a defense response against pathogens. How this is achieved is only partially understood.

AE pathogens employ a type three secretion system (TTSS) to translocate dozens of effector proteins into the host cell. These effectors subvert host cell processes, promoting colonization and formation of AE lesions [2,4]. The genes encoding for the TTSS machinery and related proteins are clustered on a pathogenicity island, 35 kb in size, termed the Locus of Enterocyte Effacement (LEE) [5]. This locus encodes for intimin and for the translocated intimin receptor (Tir), which together form the typical pathogen-cell adhesion pattern named “intimate attachment”, characteristic of AE pathogens. Additional effectors are encoded by other pathogenicity islands and prophages. These include NleC and NleD, both metalloproteases that specifically and efficiently cleave and inactivate NF-κB and MAPK, respectively [6–10], NleH, which represses transcription of a subset of NF-κB target genes [11], NleE, which inhibits activation of NF-κB signaling pathways through blocking TAK1 activation, and NleB, which catalyzes GlcNAcylation of a specific subset of Death Domains causing modest inhibition of TNF-mediated NF-κB activation, but robust inhibition of TNF-induced cell death [12–14].

In this report we show that, while infection with wild type EPEC leads to repression of NF-κB signaling, strong NF-κB activation in cells infected with an EPEC mutant deleted of the *nleB*, *nleE*, *nleC* and *nleD* effector genes was observed. Furthermore, we report that this NF-κB activation is TTSS-dependent. Investigation of the basis for this activation suggests that epithelial cells can sense the active TTSS apparatus *per se* and respond by triggering a novel signaling pathway, resulting in NF-κB activation.

## Results

### EPEC activate NF-κB by a TTSS-dependent pathway

**Fig 1:**
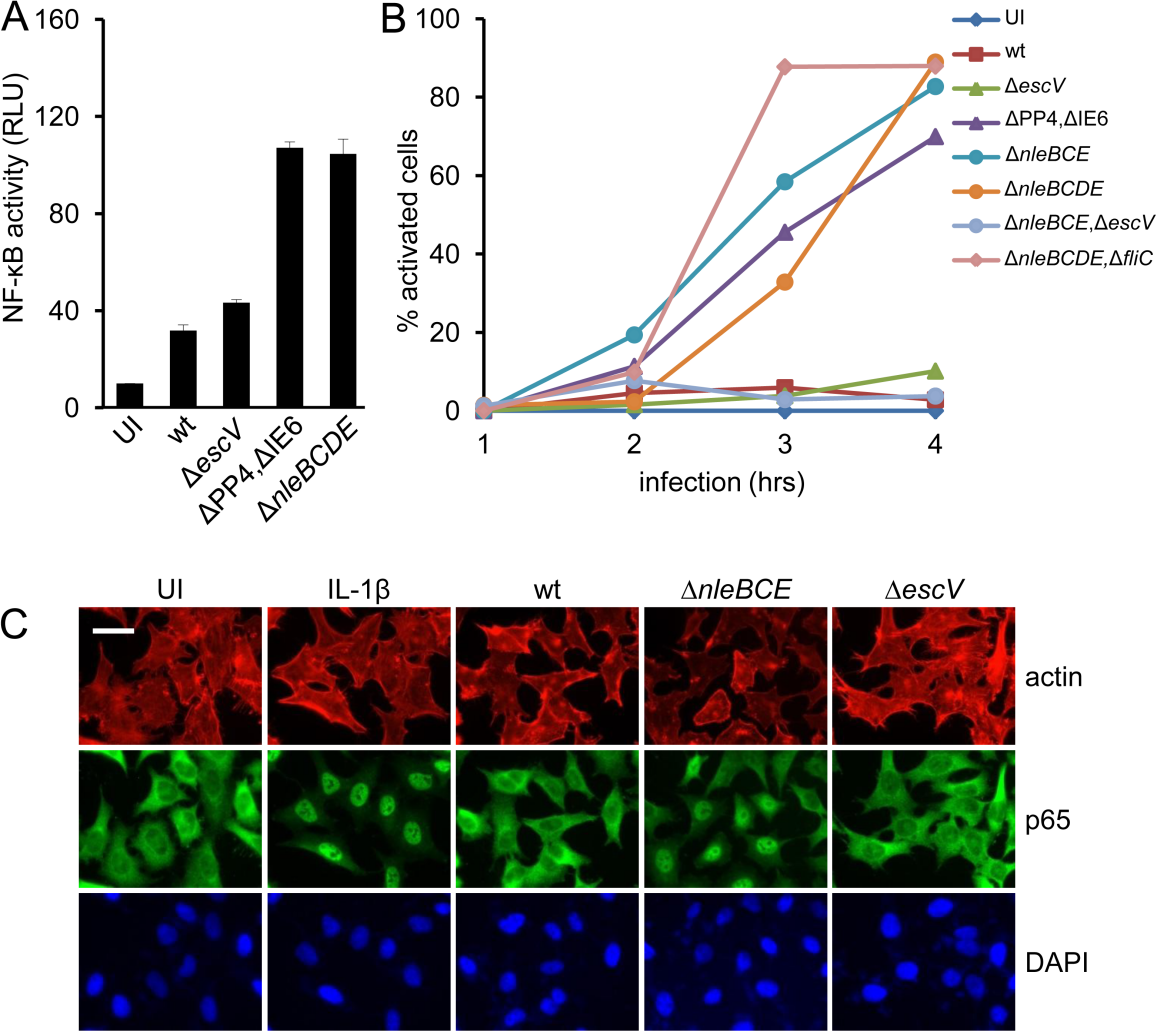
EPEC activate NF-κB by a TTSS-dependent pathway. (A) HEK293 cells were transfected with an NF-κB reporter plasmid and infected with the indicated EPEC strains or left uninfected (UI). NF-κB activation was determined using the dual-luciferase assay. Error bars represent standard deviation. (B) and (C) HeLa cells were infected with different EPEC strains, as indicated in (B), or remained uninfected (UI). Cells were fixed at the indicated time points post infection, stained for p65 (green), F-actin (red) and DNA (blue) and analyzed by fluorescent microscopy for p65 translocation to the nucleus. The percentage of activated cells is shown in (B). Error bars were omitted for clarity. Representative images from this analysis (3 hours post infection) are shown in (C). Cells treated with IL-1β were used as positive control. Size-bar represents 50 microns.

We previously reported that HeLa cells infected with an EPEC Δ*nleB* Δ*nleC* Δ*nleD* Δ*nleE* mutant (Δ*nleBCDE)* secrete fourfold more IL-8 than cells infected with EPEC mutants lacking active TTSS (i.e. *escV*::*kan* mutant) [6]. These results imply that in wild type EPEC some combination of the activities of NleB, NleC, NleD and NleE masks the capacity of EPEC to activate NF-κB. To test this idea, HEK293 cells containing a luciferase reporter for NF-κB activity were infected with different EPEC strains and luciferase activity was determined (Fig 1A). The results showed modest NF-κB activation upon infection with wild type EPEC, or EPEC lacking active TTSS (Δ*escV*), whereas the Δ*nleBCDE* mutant or a mutant deleted of the pathogenicity islands containing these genes (i.e. PP4 and IE6) triggered significantly increased NF-κB activation (p<0.01) (Fig 1A). As an additional readout for NF-κB activation, we tested translocation of the NF-κB subunit p65 upon infection from the cytoplasm to the nucleus. We found that p65 translocation was induced by EPEC Δ*nleBCDE*, or Δ*nleB*,Δ*nleC*,Δ*nleE* (Δ*nleBCE*), or Δ*PP4*,Δ*IE6* mutants (Fig 1B and 1C). Infection with a Δ*nleBCDE* mutant lacking also *fliC* (encoding flagellin) triggered similar NF-κB activation (Fig 1B), indicating that flagellin is not required for this activation. In contrast, activation was not observed upon infection with the EPEC wild type, the *escV*::*kan* mutant, or the EPEC Δ*nleBCE*,*escV*::*kan* mutant (Fig 1B). Taken together, these results show that deletion of three anti-inflammatory effectors, *nleB*, *nleC*, and *nleE* reveals NF-κB inducing activity by EPEC, which is dependent on expression of an active TTSS.

### NF-κB activation by EPEC TTSS is not dependent on effectors or flagellin

**Fig 2:**
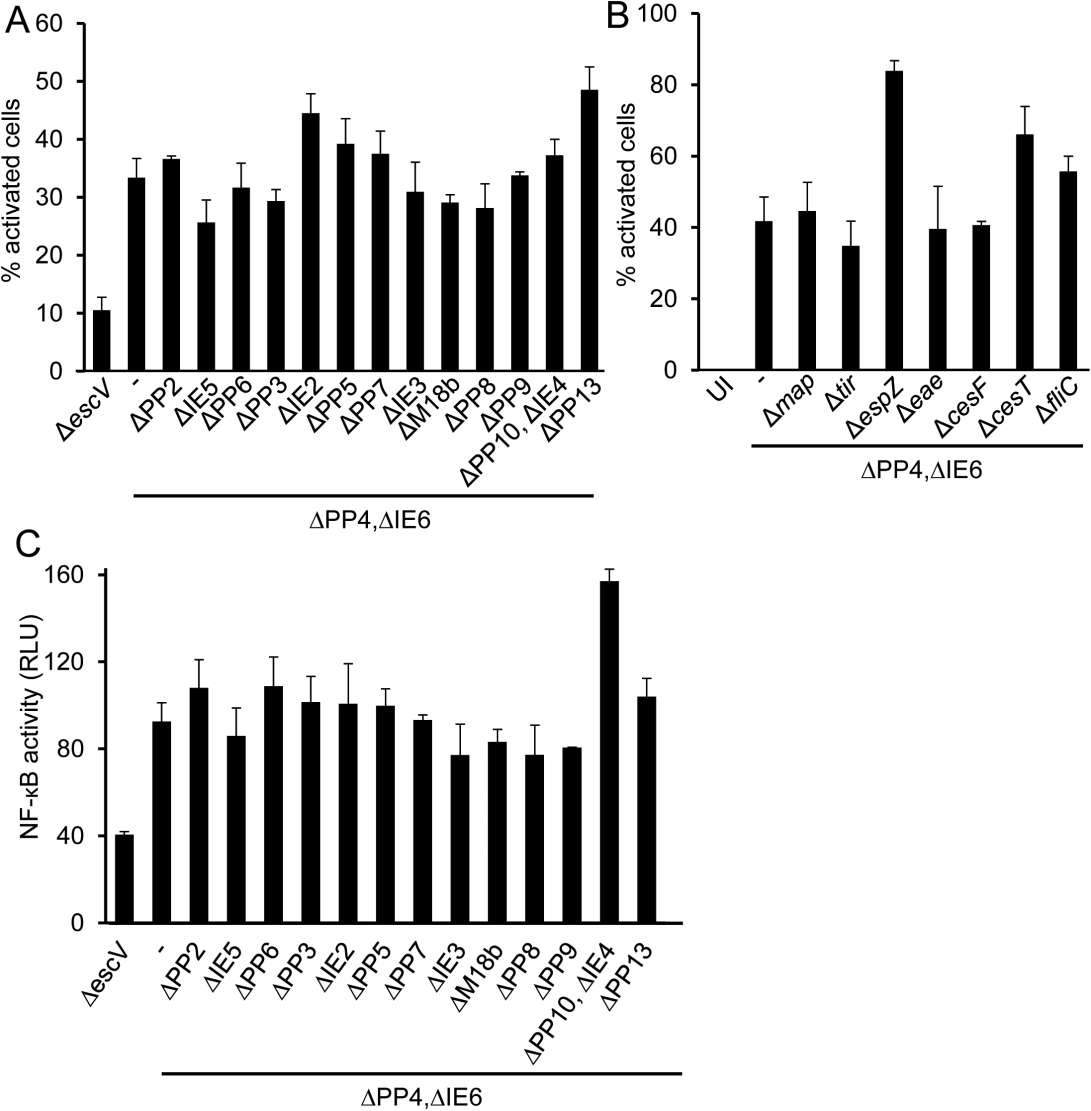
EPEC effectors are not required for NF-κB activation. HeLa cells were infected with EPEC mutants lacking *escV*, or the indicated pathogenicity islands (A), or the indicated LEE-encoded effectors genes, or the flagellin gene (*fliC*) (B). Cells were fixed and p65 translocation was analyzed by fluorescent microscopy. (C) HEK293 cells were transfected with an NF-κB reporter plasmid and infected with the indicated EPEC strains. NF-κB activation was determined using the dual-luciferase assay. Error bars represent standard deviation.

The requirement for a functional TTSS for EPEC-induced NF-κB activation suggests that this activation might be mediated by an injected effector. To identify the involved putative effector, we constructed a panel of mutants using the EPEC *ΔPP4*,*ΔIE6* mutant as a parental strain. Each mutant in this panel was deleted of a different effector gene (*tir*, *map* and *espZ*), a TTSS chaperone gene (*cesF*, *cesT*), or an entire pathogenicity island, as indicated, or other genes including *eae* (encoding intimin) and *fliC*. We performed infection assays using the above mutants and tested for p65 translocation to the nucleus and for induction of NF-κB-dependent expression. Notably, our results show that all the tested triple mutants activated NF-κB similarly to the parental strain (Fig 2A–2C), indicating that none of the genes located within the pathogenicity island or known effectors are essential in order to elicit the observed NF-κB activation.

### The TTSS apparatus is sufficient for NF-κB activation

**Fig 3:**
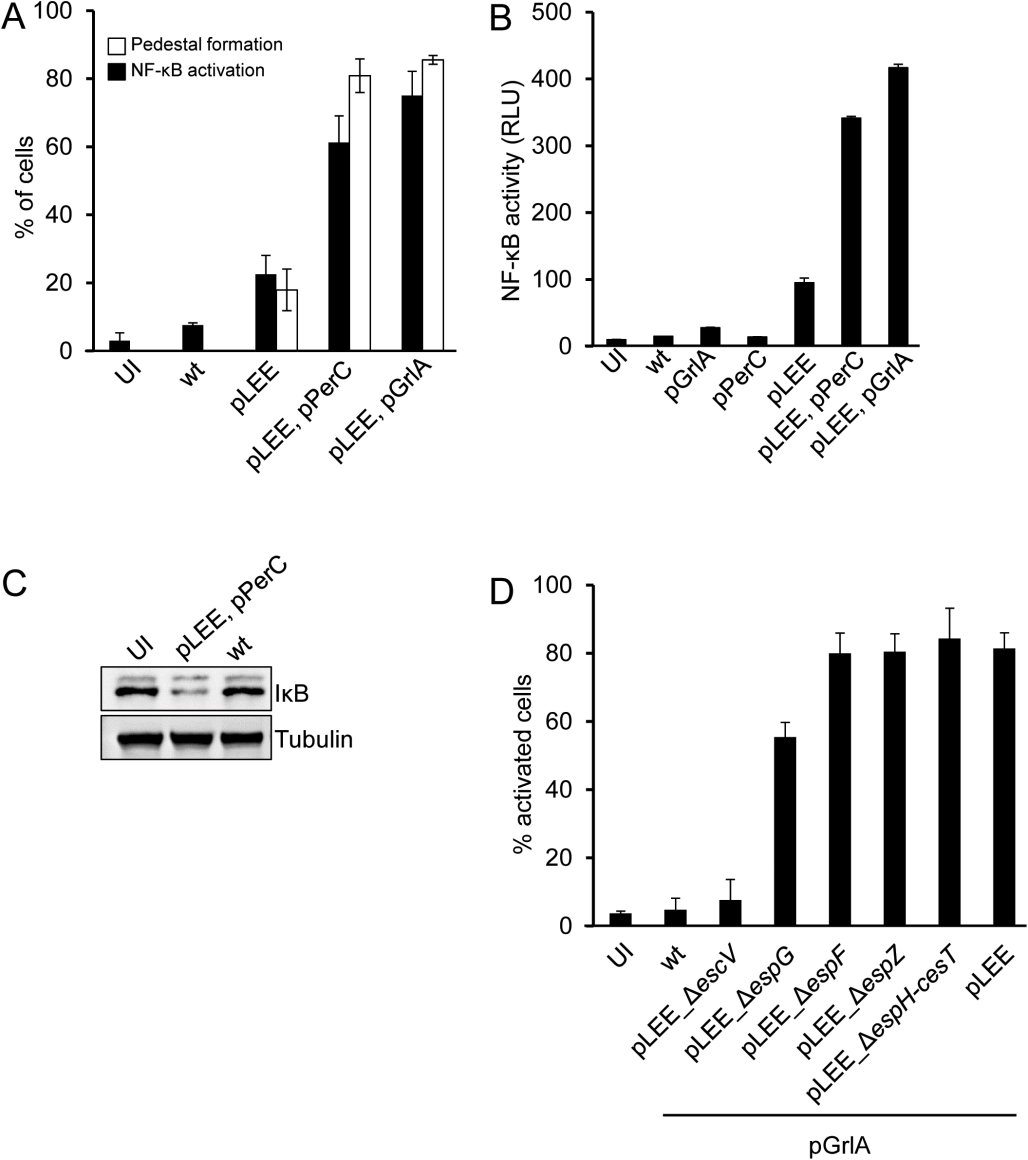
Expression of the TTSS apparatus is sufficient for NF-κB activation. (A) HeLa cells were infected for 3 hours with W3110 (wt), or with W3110 carrying different plasmids, as indicated, or remained uninfected (UI), fixed and stained for p65, F-actin and DNA (DAPI). Cells were quantified for nuclear p65 and formation of actin pedestals by fluorescent microscopy. Percentage of cells exhibiting actin pedestals and nuclear NF-κB are shown. (B) HEK293 cells transfected with the NF-κB luciferase reporter were infected with wild type W3110 (wt), W3110 carrying different plasmids, as indicated, or left uninfected (UI). NF-κB activation was determined by the dual luciferase assay. (C) HeLa cells were infected with different W3110 strains, proteins were extracted and the levels of cytosolic IκB were determined by Western blot using anti-IκB. (D) HeLa cells were infected with W3110 (wt) or W3110 carrying mutated pLEE plasmids, as indicated. Cells were fixed, stained for p65 and quantified for nuclear p65 by microscopy. Bars represent standard deviation.

The above results prompted us to examine whether the TTSS apparatus itself is sufficient for triggering NF-κB activation. To test this possibility we generated a commensal laboratory *E*. *coli* K12, strain W3110, that harbors a plasmid encoding for the entire LEE region (pTOK-O2, [8]). This strain, W3110/pLEE, produces a functional TTSS apparatus, but lacks other EPEC-unique genes, including those encoding non-LEE effectors. We infected HeLa cells with W3110/pLEE and found that it induced formation of actin pedestals in the host cells, confirming that the TTSS is functional and injects the Tir effector into host cells (Fig 3A). GrlA and PerC are known redundant positive regulators of TTSS expression [15–17]. In agreement with this, we found that expression of GrlA or PerC by W3110/pLEE strongly enhanced pedestal formation (Fig 3A). Importantly, W3110/pLEE strains, but not wild type W3110, induced p65 translocation to the nucleus (p<0.01), IκB degradation and NF-κB-dependent gene expression (p<0.01) (Fig 3B and 3C). Moreover, these phenotypes were all enhanced upon up-regulation of TTSS expression by GrlA or PerC. We conclude that expression of the LEE genes is sufficient to trigger NF-κB activation in the infected cells.

To investigate whether a LEE-encoded effector is responsible for NF-κB activation, we deleted from the pLEE plasmid the genes encoding *espF*, *espG*, *espZ* effectors and the region spanning *espH* to *cesT* (Δ*espH-cesT*). The latter region includes three effector genes (*espH*, *tir and map*) (S1 Fig), and two chaperone genes, *cesF* and *cesT* (S1 Fig), which are required for translocation of the LEE-encoded EspF and EspZ and multiple none-LEE effectors. As a negative control we also constructed pLEE deleted of *escV* and thus defective in TTSS biogenesis. We found that W3110 containing any of these pLEE mutants, except pLEE-*ΔescV*, induced NF-κB activation with a comparable efficiency to that of W3110 carrying wild type pLEE (Fig 3D). Similar results were obtained upon infection of T84 cells (S2 Fig), an epithelial cell line derived from human colon carcinoma, which better mimics intestinal epithelial cells. These results show that LEE effectors, including Tir and thus intimate attachment, are not required for the TTSS-dependent NF-κB activation. Furthermore, these results indicate that the TTSS apparatus is necessary and sufficient to induce NF-κB activation.

### The host cell detects active TTSS

**Fig 4:**
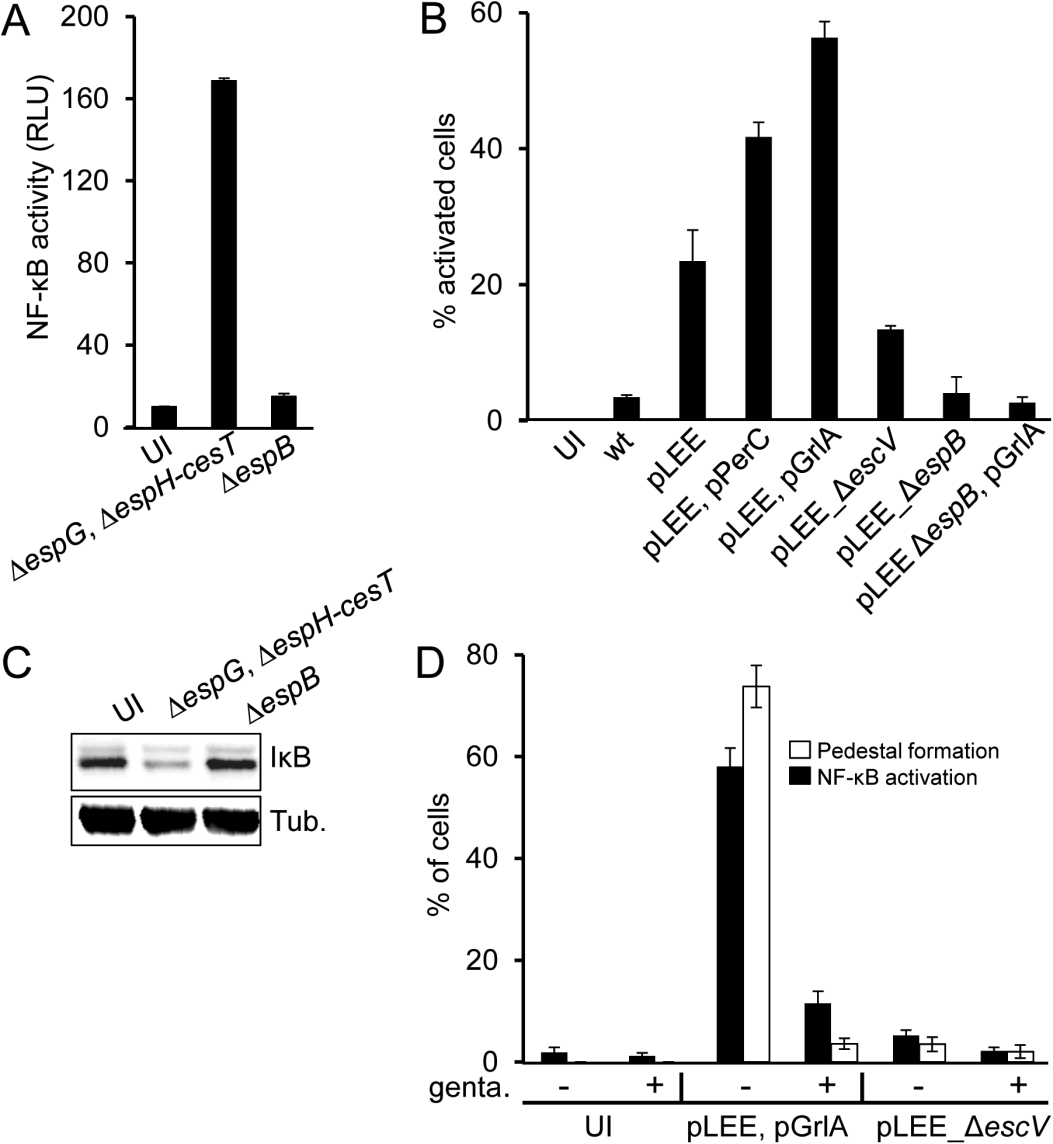
Productive contact of the TTSS with the host cell is required for NF-κB activation. (A) HEK293 cells transfected with the NF-κB reporter plasmid were infected with two W3110 strains carrying mutated pLEE plasmids, one deleted of *espG* and the region between *espH* and *cesT* (Δ*espG*, *ΔespH-cesT*), and the other deleted of *espB* (Δ*espB*). Uninfected (UI) cells were used as negative control. NF-κB activation was determined by the dual luciferase assay. (B) HeLa cells were infected with wild type W3110 (wt), or W3110 containing, as indicated, different combinations of the following plasmids: pPerC (expressing PerC [56]), pGrlA (expressing GrlA, S4 Fig) and variants of the pLEE plasmid, including wild type (pLEE) or pLEE deleted of *escV* (Δ*escV*) or *espB* (Δ*espB*). Cells were fixed, stained for p65 and nuclear p65 was quantified by microscopy. (C) HeLa cells were infected as in (B), proteins were extracted and the levels of cytosolic IκB were determined by Western blot using anti-IκB antibody. (D) W3110 containing pLEE and pGrlA, or pLEE,Δ*escV* were grown under conditions that activate TTSS formation. Were stated, gentamicin was added to block translation, during a 10 min incubation. Bacteria were then spun on HeLa cells pre-seeded on cover slips (200 g, 10 min, room temperature), and infected cells were incubated for 30 min, 37°C, 5% CO_2_. Cells were washed, fixed and stained for p65 and actin. The fraction (%) of cells with nuclear p65 and percentage of cells containing actin-pedestals was determined by microscopy.

To test if a TTSS component is sensed by a pattern recognition receptor (PRR) localized on the host cell surface, such as TLRs, we constructed two additional pLEE variants. One was deleted of the *espH-cesT* fragment as well as *espG* (S1 Fig). This mutant cannot deliver any effector into the host cell, but should still connect with the host cell by the TTSS and EspA filament [18]. The other mutant was deleted of the *espB* gene (S1 Fig). Notably, W3110/pLEE Δ*espB* still forms nearly intact TTSS, including EspA filaments, but is deficient in assembly of the TTSS pore in the host cell membrane and is thus incapable of protein translocation [19]. We found that W3110/pLEE containing Δ*espH-cesT* Δ*espG* deletions strongly activated NF-κB, whereas the W3110/pLEE Δ*espB* mutant failed to activate NF-κB (Fig 4A–4C). These results suggest that a surface PRR is not involved in NF-κB stimulation. Instead, an active pore-forming TTSS is required. To further test this notion, we took advantage of the requirement of *de-novo* protein synthesis for the activity of the EPEC TTSS [20]. For these experiments we used a different infection protocol. First, we grew W3110/pLEE under conditions that induced formation of functional TTSS, but in the absence of host cells. We then infected cells for 30 minutes with these cultures in the presence or absence of a translation inhibitor (gentamicin). This antibiotic is expected to inhibit translocation and likely reduce EPEC viability, but we assume that regardless of viability, within the 30 min infection period the molecules on the EPEC surface remain intact and capable of activating PRRs presented on the host cell surface. In the absence of gentamicin we observed efficient pedestal formation and strong NF-κB activation, while gentamicin strongly inhibited both (Fig 4D), indicating that active TTSS is required for host cell stimulation. Taken together, these results indicate that the host cell detects a functional and active TTSS by a sensor, which is not surface exposed, possibly cytoplasmic.

### The TTSS-dependent NF-κB activation is not in direct correlation with TTSS mediated pore-formation or protein translocation

TTSS activity is associated with formation of translocon pores in the host cell membrane, mediated by EspD and EspB, and with translocation of effectors through these pores [21,22]. Systematic introduction of short, in-frame, inserts into the *espB* gene lead to identification of mutations that are specifically deficient in either pore formation or protein translocation, suggesting that pore formation and protein translocation are distinct, not necessarily linked, processes [21]. We took advantage of these mutants to ask if the TTSS-dependent NF-κB activation correlates with pore formation or with protein translocation. To this aim we constructed a set of EPEC strains where the wild type *espB* gene was replaced by mutated *espB* alleles, including *espB*K179, *espB*E203, *espB*T239, *espB*L241, and *espB*L282 [21]. EPEC Δ*nleBCDE* mutant was used as a parental strain for construction of these *espB* mutants. In addition, as a negative control, we constructed EPEC Δ*nleBCDE* deleted of *espB (*Δ*espB)*. These strains are listed in S1 Table. We next used these strains to infect HeLa cells and quantified protein translocation (using pedestal formation, which is dependent on Tir translocation, as readout), pore formation (using penetration of propidium iodide (PI) into the cells as described [22]), and NF-κB activation (using p65 translocation to the nucleus as readout). We found that the pore forming activity and protein translocation by the different *espB* mutants were consistent with a previous report (compare Table 1 and [21]). In addition, the analysis showed that mutants deficient in both pore formation and protein translocation fail to activate NF-κB. These mutants carry the Δ*espB* or *espBL282* alleles, which are likely deficient in placing the EspB-EspD pore in the host cell membrane. Importantly, we could not detect direct correlation between NF-κB activation and either pore formation or protein translocation. For example, the *espB*T239 mutant showed somewhat reduced pore formation and very little protein translocation. Yet, it activated NF-κB to levels similar to those displayed by the parental strain, expressing wild type *espB* (Table 1). Furthermore, the *espB*E203 and *espB*T239 mutants induced similar levels of pore formation, yet exhibited significant differences in NF-κB activation (Table 1). Finally, the *espB*K179 mutant shows markedly reduced pore-formation, but efficient protein translocation, yet, it shows clear reduction in NF-κB activation (Table 1). Taken together, these results show that neither efficient protein translocation, nor membrane pore *per se*, is important for the TTSS-dependent NF-κB activation. Therefore, it appears that the host cells detect a cue specific to the TTSS pore, possibly some structural element of EspB.

**Table 1:** TTSS activities in EPEC strains expressing mutated EspB.

Genotype of infecting EPEC mutant	Pore-formation*	Protein translocation**	NF-κB activation***
**Δ*nleBECD*, *espB wild type***	100 (4.65)	88 (1)	40 (3)
**Δ*nleBECD*, Δ*espB***	14 (0.76)	0 (0)	2 (1)
**Δ*nleBECD*, *espB-K179***	31 (4.12)	81 (1)	17 (3)
**Δ*nleBECD*, *espB-E203***	66 (4.5)	88 (1)	11 (2)
**Δ*nleBECD*, *espB-T239***	59 (6.2)	28 (2)	45 (5)
**Δ*nleBECD*, *espB-L241***	54 (7.7)	66 (2)	50 (4)
**Δ*nleBECD*, *espB-L282***	15 (1.2)	0 (0)	10 (3)
****Uninfected cells****	16 (2.7)	0 (0)	0 (0)

HeLa cells were infected with pre-activated EPEC cultures for 30 min (pore forming assay) or one hour (protein translocation and NF-κB activation assays). Three TTSS-related activities were quantified; pore-forming activity, protein translocation and NF-κB activation. Penetration of PI into infected cells was used to determine pore-forming activity, formation of actin pedestals was used as readout for Tir translocation and localization of p65 in the nucleus was used to quantify NF-κB activation. Shown are the average values of three experiment and standard error in the parentheses.

* Relative to wild type, which set as 100.

** Percent of cells with five or more pedestals. Average of 15 different fields is shown, n > 150 cells.

*** Percent of activated cells. Average number of 15 fields is shown, n > 150 cells.

### Single TTSS components do not activate NF-κB

**Fig 5:**
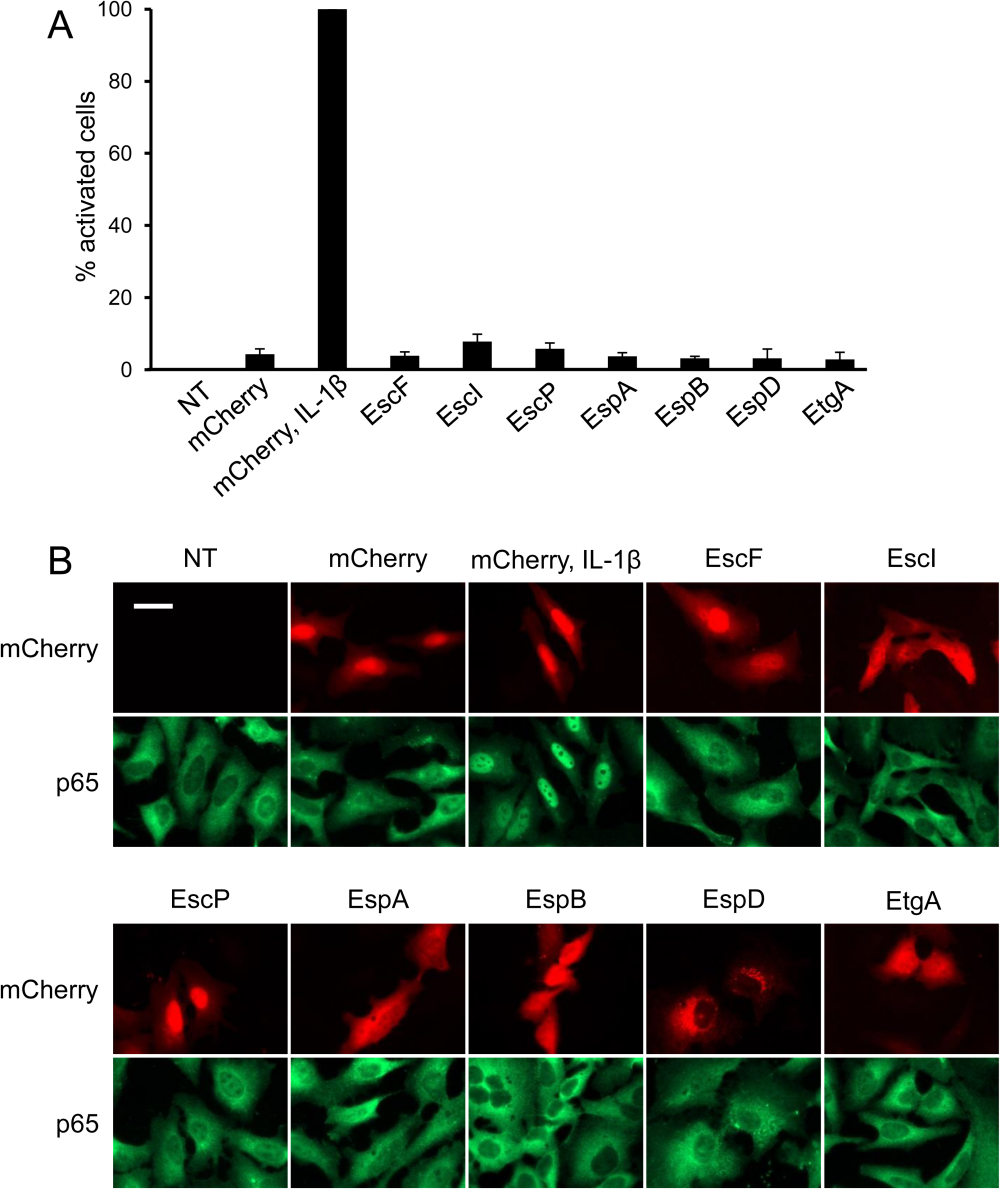
Single components of the TTSS do not trigger NF-κB activation. (A) HeLa cells were transfected with plasmids expressing mCherry-conjugants of the indicated bacterial proteins. Cells were stained for p65 translocation and quantified by microscopy. Representative images are shown in (B). Cells treated with IL-1β were used as positive control for p65 activation. Size-bar represents 50 microns.

We next examined whether a single TTSS component present inside the host cell is capable of stimulating NF-κB activation. To this end, we transfected HeLa cells with plasmids expressing different TTSS components, EscF, EscI, EscP, EspA, EspB, EspD and EtgA, which might leak into the host cell as a result of TTSS activity. We then tested whether expression of these proteins results in NF-κB activation. Notably, none of the tested TTSS components activated NF-κB (Fig 5A and 5B). These results suggest that, rather than a specific single TTSS component, the host likely senses other events associated with TTSS activity, such as the EspBD channale, membrane perturbation or leakage of non-proteinaceous components through the TTSS syringe.

### TTSS activation of NF-κB is MyD88-independent

**Fig 6:**
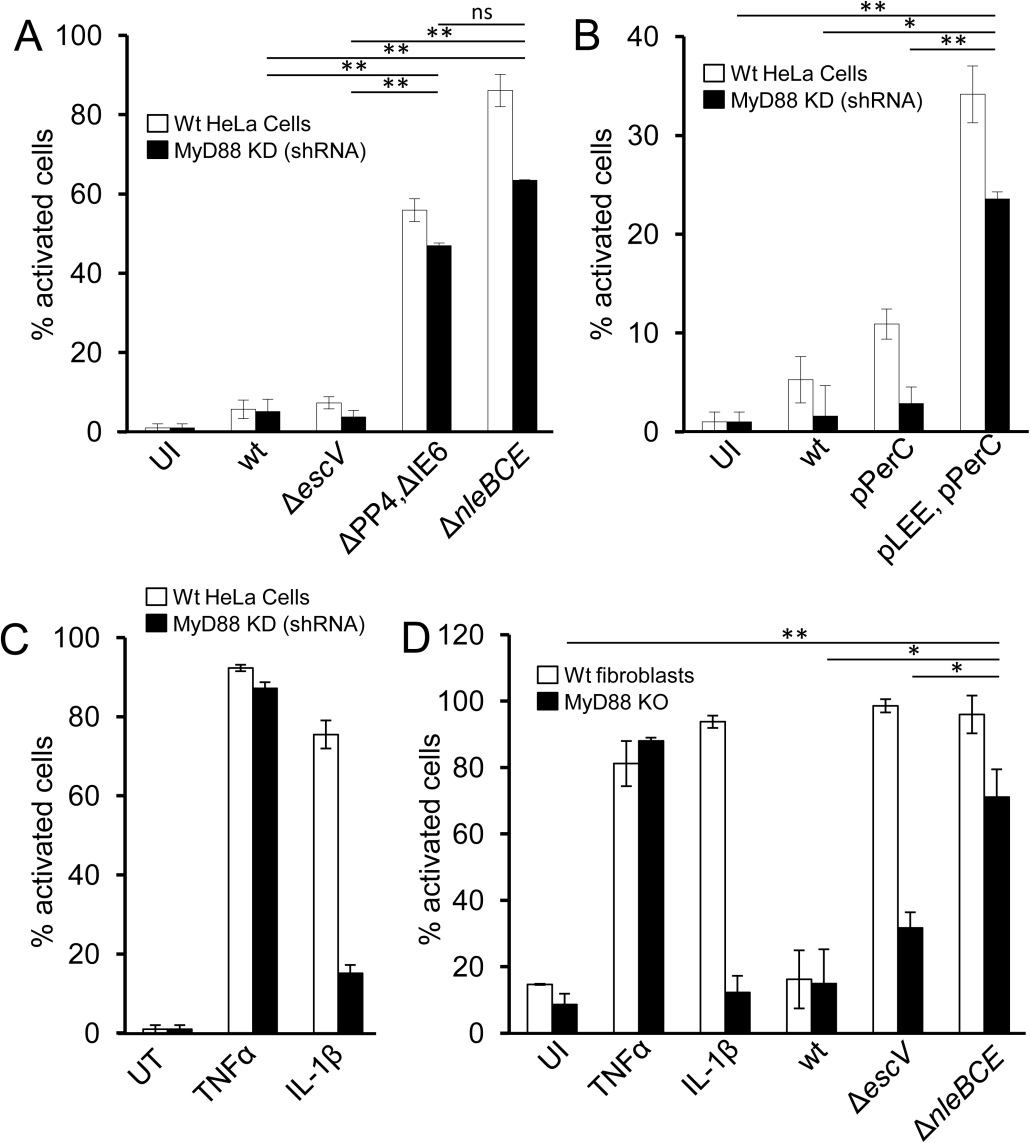
TTSS-mediated NF-κB activation is not dependent on MyD88. HeLa cells stably expressing MyD88-targeting shRNA or the control shRNA (marked as wt) were infected with EPEC (A) or W3100/pLEE (B) strains or treated with TNFα or IL-1β (C), as indicated. (D) Primary fibroblasts were extracted from wt or MyD88 knock-out C57Bl/6 mice and infected with EPEC strains or treated with TNFα or IL-1β. The cells were fixed, stained for p65 and quantified for nuclear p65 by microscopy. Bars represent standard deviation. Data are presented as mean±SE. Asterisks indicate a significant difference (*P < 0.05, **P < 0.01) using Student’s t test.

To gain better understanding of the TTSS-sensing mechanism, we searched for host factors that are involved in sensing the cue generated by TTSS activity. First we tested the requirement for MyD88, which is central to multiple TLRs as well as IL-1 signaling pathways that lead to NF-κB activation. We infected HeLa cells, stably expressing anti-MyD88 shRNA and thus deficient in MyD88 production (S3 Fig), with EPEC or W3110/pLEE. We found that both strains induced NF-κB (Fig 6A and 6B). To further control for loss of MyD88-dependent signaling, we treated these cells with TNFα or IL-1β. As expected, TNFα, but not IL-1β, activated these cells (Fig 6C) [23], confirming the functional knock-down of MyD88. Similar results were obtained using primary fibroblasts cultured from MyD88^-/-^ mice (Fig 6D). Unlike HeLa and HEK293 cells lines, we noted that primary mouse fibroblasts display MyD88-dependent NF-κB activation even upon infection with the EPEC *escV* mutant. This probably reflects expression of some TLRs that are triggered by EPEC PAMPs such as LPS or flagellin (e.g. TLR4, TLR5), but this signaling is inhibited by TTSS effectors upon infection with the wt EPEC, but not by the TTSS-deficient *escV* mutant. Nevertheless, in MyD88^-/-^ primary fibroblasts, robust NF-κB activation was revealed upon infection with the EPEC *nleB nleC nleE* mutant, but not with the *escV* mutant (Fig 6D). Taken together, these results show that MyD88 is not required for TTSS-dependent NF-κB activation.

### TRAF6 and RIP2 are dispensable for TTSS-dependent activation of NF-κB

**Fig 7:**
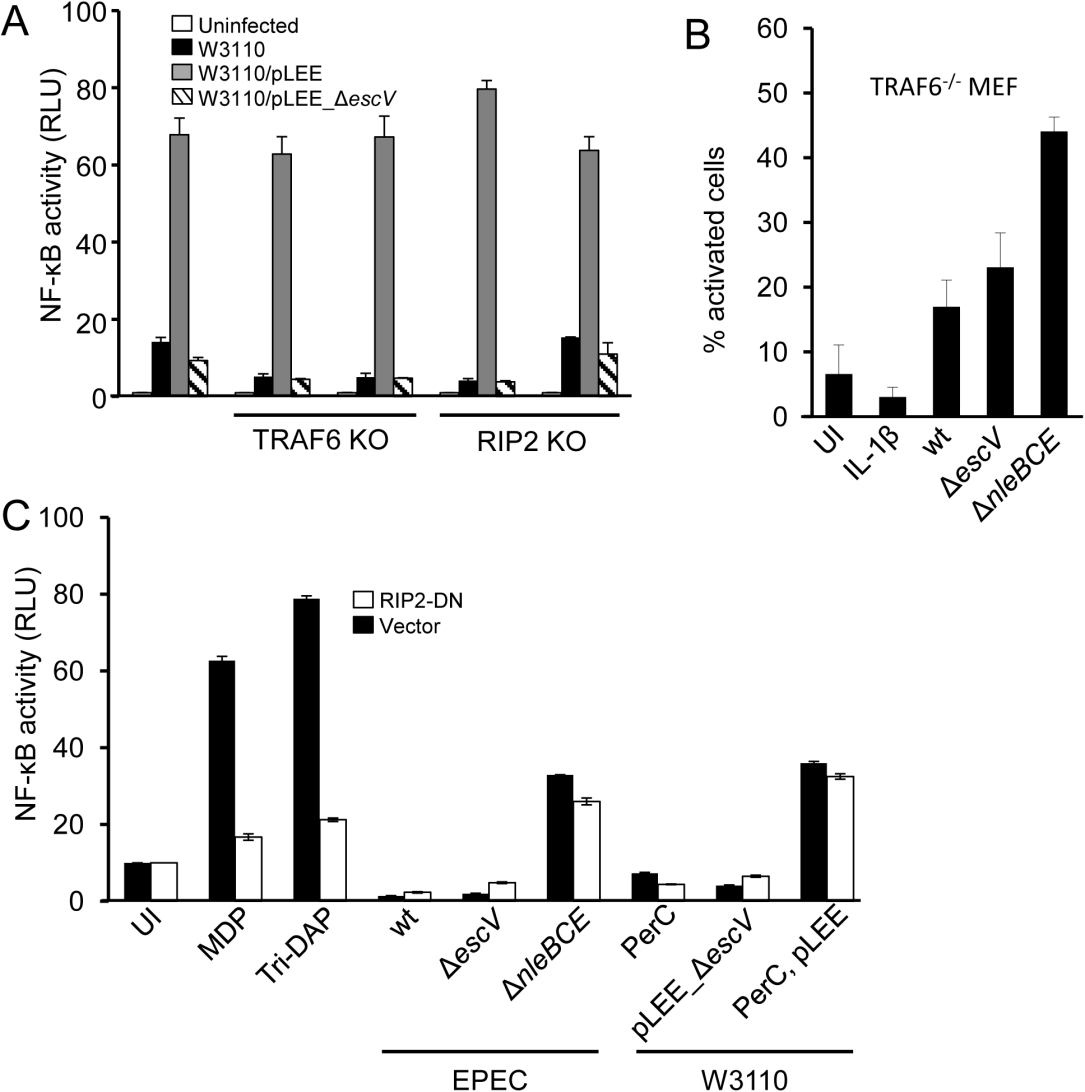
TTSS-mediated NF-κB activation is not dependent on TRAF6 or RIP2. (A) TRAF6^-/-^ or RIP2^-/-^ or wt HEK293 cells were transfected with the NF-κB reporter plasmid and infected with W3110 strains, as indicated. NF-κB activation was determined by the dual luciferase assay. Two independent clones of TRAF6^-/-^ and RIP2^-/-^ were tested. (B) TRAF6^-/-^ mouse embryonic fibroblasts were treated with IL-1β, or infected with different EPEC strains, as indicated. The cells were then fixed, stained for p65 and quantified for nuclear p65 by microscopy. (C) HEK393 cells co-transfected with the NF-κB reporter plasmid and a plasmid expressing dominant negative RIP2 (DN-RIP2) were treated with MDP, tri-DAP, or infected with EPEC or W3110 strains, as indicated. NF-κB activation was determined by the luciferase assay.

RIP2K and TRAF6 are essential components in various signaling pathways that lead to NF-κB activation [24,25]. We therefore investigated their involvement in TTSS-sensing. We generated RIP2^-/-^ and TRAF6^-/-^ HEK293 cells (see Materials and Methods). To functionally confirm the RIP2^-/-^ and TRAF6^-/-^ knockouts, the cells were transfected with plasmids overexpressing Nod1 or MyD88, respectively. As expected, Nod1 overexpression did not specifically activate NF-κB in the RIP2K^-/-^ cells, while MyD88 overexpression did not specifically activate NF-κB in the TRAF6^-/-^ cells (S5A and S5B Fig). We infected these cell lines with either W3110, W3110/pLEE or W3110/pLEE Δ*escV*, and found that W3110/pLEE, but not W3110 or W3110/pLEE Δ*escV*, induced NF-κB activation (Fig 7A). These results indicate that TRAF6 and RIP2 are not required for TTSS-mediated NF-κB activation. We further infected TRAF6^-/-^ mouse embryonic fibroblasts (MEFs, a gift from Dr. Kate Fitzgerald, University of Massachusetts) with EPEC strains and found that the EPEC Δ*nleBCE* mutant triggered NF-κB activation (Fig 7B). Finally, we found that dominant negative RIP2 (RIP2-DN) failed to inhibit TTSS-mediated NF-κB activation (Fig 7C). These results show that TRAF6 and RIP2 are not required for TTSS-dependent activation of NF-κB, reinforcing the premise that TTSS recognition is not mediated by TRAF6/MyD88-dependent signaling, or by the Nod1/2-RIP2 pathway, which is involved in peptidoglycan and ER stress sensing [26,27].

### NleE and OspI inhibit TTSS-dependent NF-κB activation

**Fig 8:**
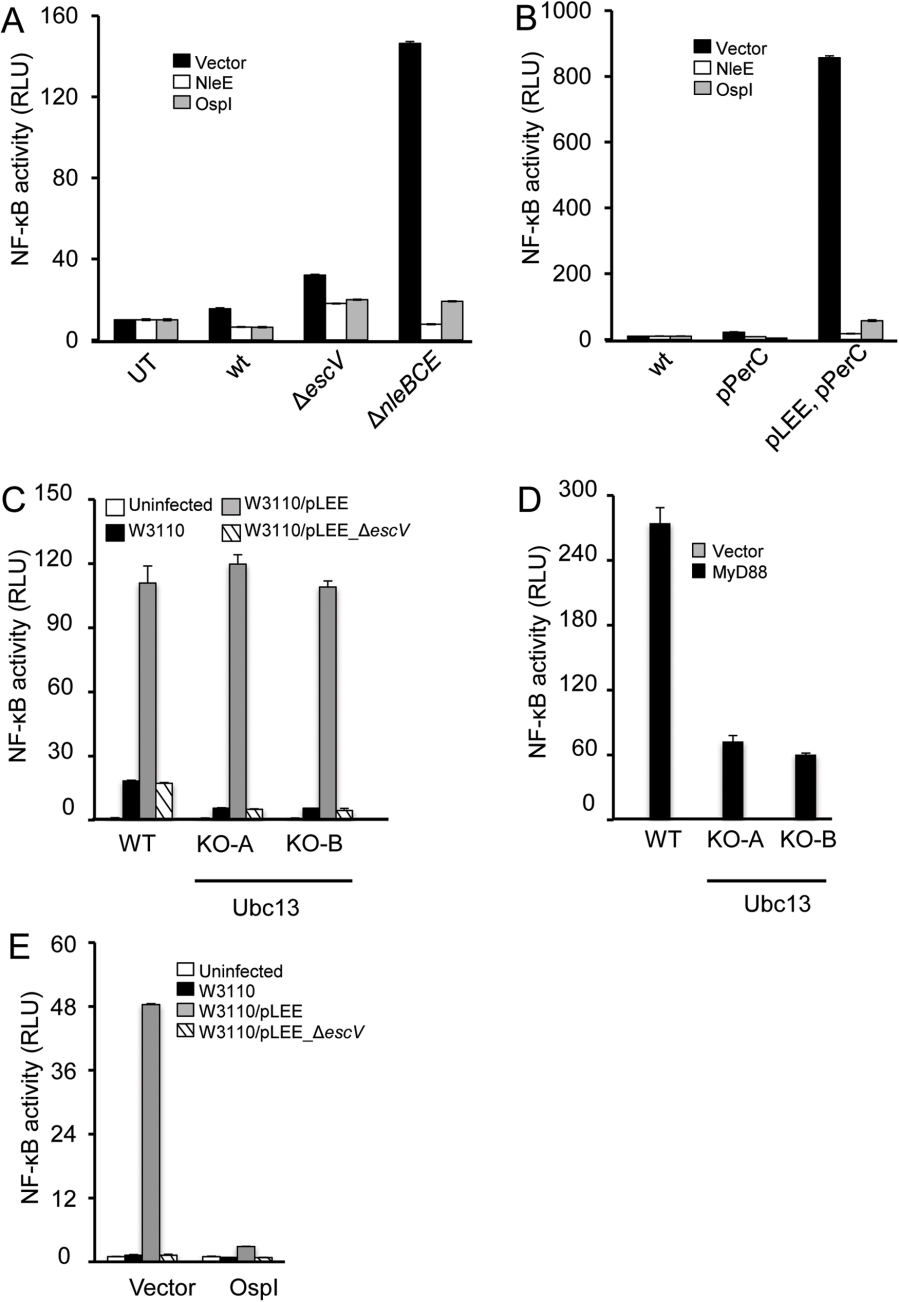
NleE and OspI inhibit TTSS-dependent NF-κB activation. HEK293 cells transfected with an NF-κB reporter plasmid and plasmids expressing mCherry-OspI, or mCherry-NleE, or vector (mCherry), were infected with EPEC (A), or W3110 strains (B). NF-κB activation was determined by the dual luciferase assay. Wild type or UBC13^-/-^ HEK293 cells were transfected with an NF-κB reporter plasmid and infected with W3110 strains as indicated (C) or co-transfected with the NF-κB reporter plasmid and a plasmid encoding MyD88, or an empty vector (D). NF-κB activation was determined by the luciferase assay. Two independent clones of UBC13^-/-^ were tested. (E) HEK293 Ubc13^-/-^ cells were co-transfected with an NF-κB reporter plasmid and a plasmid encoding OspI or an empty vector, and infected with the indicated W3110 strains. NF-κB activation was determined by the luciferase assay.

NF-κB activation frequently involves formation of K63-linked ubiquitin chains catalyzed by the E2 enzyme Ubc13. These ubiquitin chains serve as binding sites for the TAB2/3 components of the TAK1 complex, leading to TAK1 activation and subsequent NF-κB activation [28]. The *Shigella* effector OspI and the EPEC effector NleE inhibit NF-κB activation through Ubc13 deamination, and TAB2/3 methylation, respectively [14,29]. We used these effectors as tools to examine whether Ubc13 or TAK1 are involved in the signaling that leads to TTSS-dependent NF-κB activation. HEK293 cells were transfected with plasmids expressing mCherry-OspI, mCherry-NleE, or a vector expressing mCherry and were then infected with EPEC or W3100 strains. We found that expression of OspI or NleE strongly inhibited TTSS-dependent NF-κB activation (Fig 8A and 8B). These results suggest that OspI and NleE are each sufficient to block TTSS-dependent signaling that leads to NF-κB activation.

### Ubc13 is not required for NF-B activation upon TTSS sensing

Given that OspI inactivates Ubc13 [29], we assumed that Ubc13 is required for TTSS-sensing by the host cell. To test this prediction, we generated Ubc13^-/-^ HEK293 cells and infected them with W3110, W3110/pLEE or W3110/pLEE Δ*escV*. Unexpectedly, we found that W3110/pLEE induced NF-κB activation in the absence of Ubc13 (Fig 8C). In contrast, we showed that overexpression of MyD88 failed to specifically activate NF-κB in the Ubc13^-/-^ cells, confirming the lack of Ubc13 functionality (Fig 8D). Two alternative hypotheses may explain these results: *i*) OspI acts on an additional putative target, possibly another E2 enzyme that is required for TTSS-dependent NF-κB activation, or *ii*) the deaminated Ubc13 functions as a dominant negative form, which blocks TTSS-mediated NF-κB activation by stimulation of a deubiquitinating enzyme, as previously shown [30]. If the second possibility is correct, OspI should not inhibit TTSS-dependent NF-κB activation in the Ubc13^-/-^ cells. To examine this point we transfected Ubc13-/- cells with a plasmid expressing OspI, followed by infection with W3110, W3110/pLEE or W3110/pLEE Δ*escV*. The results show that in these Ubc13^-/-^ cells OspI also inhibits TTSS-mediated NF-κB activation (Fig 8E). These data exclude the possibility that deaminated Ubc13 functions as dominant negative and indicate that OspI may act on an alternative target to inhibit TTSS-dependent NF-κB activation.

## Discussion

Cells of the immune system employ an array of PRRs to detect minute amounts of MAMPs, resulting in a rapid and substantial inflammatory response, which includes activation of the NF-κB and MAPK pathways [31]. In contrast, the response of intestinal epithelial cells to the massive amounts of microbiota-derived MAMPs deposited on their apical surface must be restrained to allow colonization of the beneficial commensal microbiota and to avoid chronic inflammation [32]. This phenomenon was termed tolerance [33]. Epithelial tissues of other none-sterile mucosal source, such vaginal or nasal, should exhibit similar properties. In addition to tolerance, epithelial cells should detect small amounts of infiltrating pathogens within the enormous “noise” generated by the microbiota’s MAMPs. Rapid reaction against pathogens is advantageous for controlling the infection, but erroneous recognition of normal resident microbes as pathogens my lead to inflammatory disease [34]. The way the epithelium differentiates normal microbiota from pathogens is not fully understood and is likely to involve multiple mechanisms [32,35]. In this study we show that epithelial cells, including HeLa, HEK293 and T84, sense a functional TTSS, a virulence factor common to many gram-negative pathogens. Our findings suggest that TTSS-recognition is one of the mechanisms by which epithelial cells differentiate commensal from pathogenic bacteria, even among strains of the same species, such as commensal and pathogenic *E*. *coli* strains.

Activation of NF-κB upon TTSS recognition was previously tested for *Salmonella* and *Yersinia*, with opposite results. *Salmonella* expressing functional SPI-1 TTSS (TTSS_SPI-I_), but lacking several of the known effectors, failed to induce NF-κB in infected epithelial cells [36]. Furthermore, the ability to induce NF-κB by this pathogen was dependent on injection of specific effectors by TTSS_SPI-I_ [36,37]. Notably, more recent reports identified several TTSS_SPI-I_ anti-NF-κB effectors in *Salmonella* [38–40]. It is possible, but has never been proven, that, like in the case of TTSS_EPEC_, these effectors mask an inherent capacity of the TTSS_SPI-I_ to activate NF-κB. In contrast to *Salmonella*, a *Yersinia* mutant lacking the six known effector genes activated NF-κB in macrophages through the presence of the TTSS_Yersinia_ apparatus [41]. However, the presence of an additional effector in the *Yersinia* genome that might have NF-κB stimulatory activity cannot be excluded. Indeed, a more recent report suggests that translocation of unknown TTSS cargo leads to this activation [42]. Further studies are needed to determine if TTSS recognition is species-specific, or even specific to the TTSS-type, for pathogens that carry two or more TTSSs.

Several lines of evidence suggest that surface PRRs, such as TLRs, are not involved in TTSS sensing by epithelial cells: *i*) TTSS-mediated NF-κB activation was not dependent on MyD88 or TRAF6, which are required for TLR-mediated signalling; *ii*) EPEC, or TTSS-expressing *E*. *coli* K12 that carry *espB* deletion, fail to induce NF-κB, although these bacteria produce an almost complete TTSS apparatus; *iii*) blocking the ability of intact TTSS to translocate effectors by treatment with a translation inhibitor resulted in strong inhibition of both pedestal formation (a readout for TTSS protein translocation activity) and NF-κB activation. Collectively, our results show that epithelial cells sense only active, pore forming TTSS. Previous work has shown that the inflammasome complex, another arm of the innate immune response expressed in myeloid cells, is activated upon binding of TTSS inner rod or needle proteins to NAIP (NLR family, apoptosis inhibitory protein) cytoplasmic sensors [43]. Our work suggests that epithelial cells might utilize a cytoplasmic or membrane-embedded sensor to detect an active TTSS and trigger the NF-κB pathway. The signalling cascade induced upon TTSS-recognition is inhibited by NleE and OspI, suggesting that the TTSS-mediated pathway converges with other signalling pathways that lead to NF-κB activation at the level of binding of the TAB2/3-TAK1 complex to K63-linked ubiquitin chains, resulting in TAK1 activation.

NF-κB activating effectors were described for several pathogens, including *Salmonella* and rare EPEC isolates [36,37,44]. We therefore initially examined the hypothesis that EPEC activates NF-κB through injection of a specific pro-NF-κB effector. However, extensive analysis, using several complementing approaches, indicated that no known effector of EPEC is required for TTSS-mediated NF-κB activation. Therefore, our results suggest that NF-κB activation is mediated by a TTSS-related cue, but is independent of protein translocation or a specific effector. Furthermore, our data using different *espB* mutants show that the pore-forming *per se* is not sufficient to elicit NF-κB activation. Thus, the host specifically detects the active TTSS. The precise cue that is sensed by host cells is yet to be defined. It might be a structural element of the pore, possibly some EspB structure. Alternatively, a common metabolite, such as monosaccharide heptose-1,7-bisphosphate (HBP) [45], or peptidoglycan (PG) products [46], might leak into the host cell through the TTSS and be sensed by the host. HBP and PG are not likely to be related to the TTSS-dependent activation since they are dependent on TRAF6 and RIP2, respectively. Nevertheless, metabolite analogues of HBP or PG might be involved. An alternative possibility is that the transient membrane damage typical to TTSS might provoke signalling that leads to NF-κB activation.

AE pathogens reside mainly on the apical surface of the intestinal epithelium and inject into these cells several effectors (NleC, NleD, NleE, NleH) that repress NF-κB and MAPK signalling [6,8,9,11,14,47]. Our finding that NF-κB signalling is activated upon TTSS-recognition provides a plausible role for the anti-NF-κB effectors, i.e., to neutralize the host pro-inflammatory response mediated by TTSS-recognition. In this context, it is worth mentioning that accumulating data suggest that EPEC can establish a long-term sub-clinical carrier state in humans [48,49]. Thus, preventing or attenuating the host inflammatory response elicited by these effectors might provide EPEC with a concealed niche allowing it to establish long-term colonization. Furthermore, dampening the host response by EPEC might be involved in the increased susceptibility to secondary challenges, as was recently reported [50].

In conclusion, our results show that the host epithelial cells can detect the active, pore forming, TTSS of EPEC to trigger a novel signalling pathway, leading to NF-κB activation. Notably, EPEC acquired effectors that dampen this NF-κB activation through horizontal gene transfer.

## Material and methods

### Bacterial strains, plasmids and primers

Plasmids, bacterial strains, and primers used in this study are listed in S1–S3 Tables, respectively. Mutants of EPEC and pLEE plasmid were constructed using the lambda red system and selective cassettes [51–54]. Genomic *espB* short-insertion mutants were constructed using as templates plasmids encoding the mutants [21], lambda red system and *tet-sacB* cassette as described [51]. Plasmids were constructed using standard methods or isothermal assembly. Bacteria were grown in LB supplemented, when appropriate, with ampicillin (50 μg/ml), streptomycin (50 μg/ml), tetracycline (10 μg/ml), chloramphenicol (25 μg/ml) or kanamycin (40 μg/ml). Infection was performed by diluting an overnight standing LB culture of bacteria (1:100 for EPEC, or 1:75 for W3110 strains) with antibiotic-free DMEM.

### Infection conditions

Bacteria were grown overnight in a static LB culture, at 37°C to OD_600_ ~0.8, diluted 1:100 in antibiotic-free DMEM and applied on the cells (MOI ~100). Infections proceeded for 3 hours. Of note, initially the bacteria do not express TTSS. The transition to DMEM activates TTSS expression, which becomes fully functional after 2 hours. For luciferase assays, infection was stopped by adding gentamicin followed by additional 3-hour incubation to allow production of luciferase by the host cells. In other cases, infection was stopped by fixation, or by protein extraction. To pre-induce TTSS formation by W3110 as shown in Fig 4D, an overnight standing culture in LB was diluted 1:75 in DMEM and incubated at 37°C for 1 hour. IPTG (0.2 mM) was then added to activate TTSS expression and incubation was continued for another 2 hours.

### Cell lines

Primary fibroblasts were produced from tails of wild type and MyD88 knock-out C57BL/6 mice by incubating the tissue in a trypsin-EDTA solution for 1 hour at 37°C. HeLa cells stably expressing MyD88-targeting shRNA or control shRNA were constructed using shRNA from Sigma Inc. MEF TRAF6^-/-^ were a gift from K. Fitzgerald. Generation of HEK293 TRAF6^-/-^ and RIP2^-/-^ cell lines using the CRISPR/Cas9 method has been recently described [55]. The inactivation of the corresponding genes was verified by sequence analysis. The guide RNA (gRNA) target sequences used were CCACGCAGACTGGCGCGTCC (for RIP2 KO) and ATCTTTTGTTACAGCGCTAC (for TRAF6 KO). Cell clones with the desired gene knockout were checked by sequencing of the PCR fragments. Cells were grown in DMEM supplemented with 10% fetal calf serum, penicillin and streptomycin. When appropriate, cells were treated with TNFα (Peprotech, 300-01A), IL-1β (Biotest, 201-LB-005), or gentamicin (100 μg/ml).

### Immunofluorescence microscopy

HeLa cells were grown overnight to 70% confluence in 24-well plates and infected for the indicated time. Cells were then washed, fixed and stained with anti-p65 (SC-372, Santa Cruz), phalloidin-rhodamine (P1951, Sigma) and DAPI (D9542, Sigma), followed by washing and staining with anti-rabbit Alexa Fluor 488 conjugated antibody (#4812, Cell Signaling). Cells were visualized using fluorescence microscopy. All experiments were repeated at least three times and significance was tested using the student T-test (un-paired, two-tailed).

### Luciferase assay

The assay was performed according to the instructions in the Dual-Luciferase Reporter kit (Promega). Briefly, HEK293T cells were grown overnight in DMED media to 70% confluence on top of poly-lysine (P8920, Sigma)-coated glass cover slips in 24-well plates, co-transfected with 0.2 μg and 0.025 μg of pNF-κB-luc and pRL-TK Renilla luciferase vector (Promega) DNA, respectively, using the TurboFect transfection reagent (R0531, Fermentas). After 24 hours, the medium was replaced with fresh DMEM and the cells were infected for 3 hours with bacteria from an overnight bacteria culture at a MOI of 1:100. The medium was then supplemented with gentamicin (100 μg/μl) in order to stop bacterial replication and avoid significant cell death. Cells were incubated for another 3 hours to allow accumulation of the reporter gene expression to detectable levels. Relative luminescence units (RLU) were normalized to those of uninfected cells. All experiments were repeated at least three times and significance was tested using the student T-test (un-paired, two-tailed).

### IκB stability

HeLa cells were infected with bacterial strains for 3 hours and harvested by centrifugation. Proteins were extracted using the NE-PER kit (Thermo Scientific), analyzed by Western blot and stained with anti-IκB (#9242, Cell Signaling) and anti-tubulin (T9026, Sigma).

### Pore-forming activity

Pore-forming activity was determined as described [22], with slight modifications. Briefly, 2x10^5^ HeLa cells per well were seeded in 24 well plates (In Vitro Scientific, black plate, glass bottom). Upon reaching confluency of ~90% the cells were infected, washed with cold PBS, incubated for 2 min with 7.5 mM propidium iodide (PI) in PBS, washed twice, fixed with 2% paraformaldehyde and washed twice in PBS. The amount of PI in the cells was determined using a plate reader (SPARK-10M TECAN, monochromators set at 533nm excitation, 620nm emission).

## Supporting information

S1 Table List of plasmids used in this study. (DOCX)

S2 Table List of strains used in this study. (DOCX)

S3 Table List of primers used in this study. (DOCX)

S1 Fig EPEC type three secretion system. Upper panel: The locus of Enterocyte Effacement (LEE) is presented with color-coding depicting the nature of the genes encoded. Lower panel: Schematic diagram of the TTSS structures formed by wild type bacteria, the Δ*escV* mutant and the Δ*espB* mutant. Shown are the locations of the EspB and EscV proteins in the TTSS with respect to the bacterial inner membrane (IM), outer membrane (OM) and host cell membrane (HM).
(TIF)

S2 Fig NF-κB activation in human colonic epithelial cells. (A) T84 human colonic epithelial cells were infected with EPEC or with W3110 strains as indicated, or were treated with TNFα. The cells were then fixed, stained for p65 and quantified for nuclear p65 by microscopy. Representative images from this analysis (3 hours post infection) are shown in (B). Size-bar represents 200 microns.
(TIF)

S3 Fig Reduction in MyD88 expression in MyD88 knockdown HeLa cells. Wild-type (WT) and lentivirally transduced cells (shMyD88) were tested for MyD88 expression by qPCR and Western blot. (A) qPCR results normalized to beta-actin. (B-C) relative MyD88 protein by densitometry normalized for the amount of protein using beta actin as a control. (A) and (B) represent the average of three independent experiments. (C) One representative WB is shown.
(TIF)

S4 Fig Expression of GrlA. Bacteria containing, or not, plasmids expressing GrlA or GrlA-HA (as indicated), were treated, or not, with IPTG (as indicated). Proteins were extracted and resolved using Stain-Free^TM^ gel. After recording the amount of total proteins, the gel was used for Western blot analysis using anti HA antibody. The strains, plasmids and IPTG treatment are indicated above the lanes. Molecular size markers are shown on the right.
(TIF)

S5 Fig Verification the lack of TRAF6 or RIP2 activities in the corresponding knockout cells. TRAF6^-/-^ or RIP2^-/-^ or wt HEK293 cells were co-transfected with the NF-κB reporter plasmid and a plasmid encoding Nod1 (A) or MyD88 (B), which activate NF-κB through the RIP2 and TRAF6 pathways, respectively, or an empty vector. NF-κB activation was determined by the dual luciferase assay.
(TIF)
