# Supplementary material for: Epithelial cells detect functional type III secretion system of enteropathogenic Escherichia coli through a novel NF-κB signaling pathway
Source: PLoS Pathog. 2017 Jul 3;13(7):e1006472. doi: 10.1371/journal.ppat.1006472 (PMC5510907; doi:10.1371/journal.ppat.1006472)
Supplement: S1 Table — (DOCX) [file ppat.1006472.s001.docx]

Table S1 - List of plasmids

| **Plasmid name (number)** | **Description** | **Source** |
| --- | --- | --- |
| pTOK-02 (5574) | pTOK-02 is pCC1FOS that contains the entire LEE element of EPEC strain B171-8 O111:NM. For simplicity, this plasmid is termed pLEE. Cm^R^ | *Ref. 8* |
| 5969 | pLEE Δ*escV::tetA-sacB* constructed with primers 1699 and 1700. | *This study* |
| 6227 | pLEE Δ*espZ::tetA-sacB* constructed with primers 1824 and 1698. | *This study* |
| 5966 | pLEE Δ*espG::tetA-SacB* constructed with primers 1693 and 1694. | *This study* |
| 5967 | pLEE Δ*espF::tetA-SacB* constructed with primers 1695 and 1696. | *This study* |
| 5968 | pLEE Δ*espH-cesT::tetA-SacB* constructed with primers 1691 and 1692. | *This study* |
| 6267 | pLEE Δ*espH-cesT::tetA-sacB, ΔespG::Kan* constructed with primers 1925 and 1926. | *This study* |
| 6285 | pLEE Δ*espB::Kan* constructed with primers 1946 and 1947. | *This study* |
| pSC4141 | Eukaryotic expression vector for generation of mCherry fusions. | *Ref. 44* |
| 6196 | pSC4141 encoding *mCherry*-*escF* was constructed with primers 1852,1853,1854, and 1855 using isothermal assembly. | *This study* |
| 6197 | pSC4141 encoding *mCherry*-*escI* was constructed with primers 1852,1853,1856, and 1857 using isothermal assembly. | *This study* |
| 6198 | pSC4141 encoding *mCherry*-*escP* was constructed with primers 1852,1853,1858, and 1859 using isothermal assembly. | *This study* |
| 6199 | pSC4141 encoding *mCherry*-*espA* was constructed with primers 1852,1853,1860, and 1861 using isothermal assembly. | *This study* |
| 6200 | pSC4141 encoding *mCherry*-*espB* was constructed with primers 1852,1853,1862, and 1863 using isothermal assembly. | *This study* |
| 6201 | pSC4141 encoding *mCherry*-*espD* was constructed with primers 1852,1853,1864, and 1865 using isothermal assembly. | *This study* |
| 6202 | pSC4141 encoding *mCherry*-*espZ* was constructed with primers 1852,1853,1866, and 1867 using isothermal assembly. | *This study* |
| 6203 | pSC4141 encoding *mCherry*-*etgA* was constructed with primers 1852,1853,1868, and 1869 using isothermal assembly. | *This study* |
| 6205 | pSC4141 encoding *espZ-mCheery* was constructed with primers 1870,1871,1874, and 1875 using isothermal assembly. | *This study* |
| 6206 | pSC4141 encoding *escP-mCherry* was constructed with primers 1870,1871,1876, and 1877 using isothermal assembly. | *This study* |
| 6207 | pSC4141 encoding *escI-mCherry* was constructed with primers 1870,1871,1878, and 1879 using isothermal assembly. | *This study* |
| 6208 | pSC4141 encoding *espD-mCherry* was constructed with primers 1870,1871,1880, and 1881 using isothermal assembly. | *This study* |
| 6209 | pSC4141 encoding *etgA-mCherry* was constructed with primers 1870,1871,1882, and 1883 using isothermal assembly. | *This study* |
| 6210 | pSC4141 encoding *espA-mCherry* was constructed with primers 1870,1871,1884, and 1885 using isothermal assembly. | *This study* |
| 6211 | pSC4141 encoding *escF-mCherry* was constructed with primers 1870,1871,1886, and 1887 using isothermal assembly. | *This study* |
| 6212 | pSC4141 encoding *espB-mCherry* was constructed with primers 1870,1871,1888, and 1889 using isothermal assembly. | *This study* |
| 6204 | pSC4141 encoding *ospI*-*mCherry* was constructed with primers 1870,1871,1872, and 1873 using isothermal assembly. | *This study* |
| 4733 | pSC4141 encoding *mCherry*-*nleE* constructed with primers 1232 and 1233. | *This study* |
| 5226 | pSC4141 encoding *mCherry*-*espJ* constructed with primers 1386 and 1387. | *This study* |
| 5228 | pSC4141 encoding *mCherry*-*nleG* constructed with primers 1390 and 1391. | *This study* |
| 5229 | pSC4141 encoding *mCherry*-*nleF* constructed with primers 1392 and 1393. | *This study* |
| 5230 | pSC4141 encoding *mCherry*-*nleH2* constructed with primers 1394 and 1395. | *This study* |
| 5231 | pSC4141 encoding *mCherry*-*nleA* constructed with primers 1396 and 1397. | *This study* |
| 5233 | pSC4141 encoding *mCherry*-*espG2* constructed with primers 1400 and 1401. | *This study* |
| 5235 | pSC4141 encoding *mCherry*-*tir* constructed with primers 1404 and 1405. | *This study* |
| 5236 | pSC4141 encoding *mCherry*-*map* constructed with primers 1406 and 1407. | *This study* |
| 5238 | pSC4141 encoding *mCherry*-*espF* constructed with primers 1410 and 1411. | *This study* |
| 6259 | The pSC4141 expression vector in which the mCherry was replaced by BFP. Backbone was amplified with primers 1899 and 1900. BFP was amplified from plasmid p5923 using primers 1901 and 1902. | *This study* |
| 6260 | p6949 encoding BFP fused to a dominant-negative RIP2 assembled using primers 1899, 1900, 1901, 1903, 1904, and 1905. | *This study* |
| pRL-TK (5028) | Constitutively expressing the renilla luciferase. | *Promega (E2241)* |
| pNFkB-luc (4723) | A plasmid expressing firefly luciferase under an NF-κB-dependent promoter. | *Ref. 31* |
| 2892 | pSA10 encoding *GrlA*. | *Ref. 15* |
| 7332 | pSA10 encoding *GrlA*-HA (C'). Backbone was amplified with primers 3659 and 3660. | *This study* |
| pFT-A (4454) | Expressing flipase under an inducible tet promoter. | *Ref. 51* |
| pVCD450 (42) | A plasmid based on pACYC184, expressing *perC*. | *Ref. 17* |
| pKD3 (812) | Template for the chloramphenicol resistance cassette. | *Ref. 49* |
| pKD4 (813) | Template for the kanamycin resistance cassette. | *Ref. 49* |
| pKD46 (811) | A temperature sensitive plasmid that expresses lambda Red recombinase. | *Ref. 49* |
| pEBFP2-nuc (5923) | Used as template to amplify BFP. | *Addgene (14893)* |
| pEspB179 (7292) | Used as template to amplify *espB-K179*, harboring a 5-Codon insertion after K179 in espB. EspB amplified with primers 3678 and 3679. | *Ref. 21* |
| pEspB203 (7293) | Used as template to amplify *espB-E203*, harboring a 5-Codon insertion after E203 in espB. EspB amplified with primers 3678 and 3679. | *Ref. 21* |
| pEspB239 (7294) | Used as template to amplify *espB-T239*, harboring a 5-Codon insertion after T239 in espB. EspB amplified with primers 3678 and 3679. | *Ref. 21* |
| pEspB241 (7295) | Used as template to amplify *espB-L241*, harboring a 5-Codon insertion after L241 in espB. EspB amplified with primers 3678 and 3679. | *Ref. 21* |
| pEspB282 (7296) | Used as template to amplify *espB-K282*, harboring a 31-Codon insertion after K282 in espB. EspB amplified with primers 3678 and 3679. | *Ref. 21* |
