# Supplementary material for: Epithelial cells detect functional type III secretion system of enteropathogenic Escherichia coli through a novel NF-κB signaling pathway
Source: PLoS Pathog. 2017 Jul 3;13(7):e1006472. doi: 10.1371/journal.ppat.1006472 (PMC5510907; doi:10.1371/journal.ppat.1006472)
Supplement: S2 Table — (DOCX) [file ppat.1006472.s002.docx]

| Table S2 - List of strains | |  |  |
| --- | --- | --- | --- |
|  | **Genotype** | **Description** | **Source** |
| **EPEC derivatives** |  |  |  |
| E2348/69 | wt, O127:H7 |  | *Ref. 2* |
| SK1961 | *escV*::miniTn10*Kan* |  | *Ref .6* |
| SK4240 | ΔPP4::*Kan*, ΔIE6::*Cm* |  | *Ref. 6* |
| SK4970 | ΔPP4, ΔIE6 | SK4240 deleted of the kanamycin and chloramphenicol cassettes using the pFT-A plasmid. | *This study* |
| SK5174 | ΔPP4, ΔIE6, Δ*escV*::*Kan* | SK4970 deleted of *escV* using primers 1020 and 1021. | *This study* |
| KB4325 | ΔIE2::*Cm*, Δ*nleBE*, ΔIE6::T*et*, Δ*nleC*::*Kan* |  | *Ref. 6* |
| KB4875 | ΔIE2::*Cm*, Δ*nleBE*, ΔIE6::*Tet*, Δ*nleC* | KB4325 deleted of the kanamycin cassette using the pFT-A plasmid. | *This study* |
| KB4329 | ΔIE2::*Cm*, Δ*nleBE*, ΔIE6::*Tet*, Δ*nleCD*::*Kan* |  | *Ref. 6* |
| SK4941 | ΔIE2::*Cm*, Δ*nleBE*, ΔIE6::*Tet*, Δ*nleC*, Δ*fliC*::*Kan* | KB4875 deleted of *fliC* using primers 1330 and 1331. | *This study* |
| SK4973 | ΔPP4, ΔIE6, ΔPP2::*Kan* | SK4970 deleted of PP2 using primers 697 and 680. | *This study* |
| SK4986 | ΔPP4, ΔIE6, ΔIE5::*Kan* | SK4970 deleted of IE5 using primers 647 and 692. | *This study* |
| SK5029 | ΔPP4, ΔIE6, ΔPP6::*Kan* | SK4970 deleted of PP6 using primers 685 and 701. | *This study* |
| SK5075 | ΔPP4, ΔIE6, ΔPP3::*Kan* | SK4970 deleted of PP3 using primers 667 and 681. | *This study* |
| SK5076 | ΔPP4, ΔIE6, ΔIE2::*Kan* | SK4970 deleted of IE2 using primers 670 and 683. | *This study* |
| SK5077 | ΔPP4, ΔIE6, ΔPP5::*Kan* | SK4970 deleted of PP5 using primers 700 and 684. | *This study* |
| SK5078 | ΔPP4, ΔIE6, ΔPP7::*Kan* | SK4970 deleted of PP7 using primers 706 and 686. | *This study* |
| SK5079 | ΔPP4, ΔIE6, ΔIE3::*Kan* | SK4970 deleted of IE3 using primers 673 and 687. | *This study* |
| SK5080 | ΔPP4, ΔIE6, Δ18Mb::*Kan* | SK4970 deleted of 18Mb (see Ref. 28) using primers 676 and 688. | *This study* |
| SK5081 | ΔPP4, ΔIE6, ΔPP8::*Kan* | SK4970 deleted of PP8 using primers 709 and 689. | *This study* |
| SK5082 | ΔPP4, ΔIE6, ΔPP9::*Kan* | SK4970 deleted of PP9 using primers 712 and 690. | *This study* |
| SK5083 | ΔPP4, ΔIE6, ΔPP10::*Kan* | SK4970 deleted of PP10 using primers 715 and 691. | *This study* |
| SK5084 | ΔPP4, ΔIE6, ΔPP13::*Kan* | SK4970 deleted of PP13 using primers 718 and 694. | *This study* |
| SK5430 | ΔPP4, ΔIE6, Δ*eae*::*Kan* | SK4970 deleted of *eae* using primers 1498 and 1499. | *This study* |
| SK5431 | ΔPP4, ΔIE6, Δ*cesF*::*Kan* | SK4970 deleted of *cesF* using primers 1005 and 1006. | *This study* |
| SK5432 | ΔPP4, ΔIE6, Δ*cesT*::*Kan* | SK4970 deleted of *cesT* using primers 1007 and 1008. | *This study* |
| SK5433 | ΔPP4, ΔIE6, Δ*fliC*::*Kan* | SK4970 deleted of *fliC* using primers 942 and 943. | *This study* |
| SK5434 | ΔPP4, ΔIE6, Δ*tir*::*Kan* | SK4970 deleted of *tir* using primers 1496 and 1497. | *This study* |
| SK5566 | ΔPP4, ΔIE6, Δ*espZ*::*Kan* | SK4970 deleted of *espZ* using primers 544 and 393. | *This study* |
| KB5004 | ΔPP4, ΔIE6, Δ*map*::*Kan* | SK4970 deleted of *map* using primers 1006 and 721. | *This study* |
| SS6108 | ΔnleB, ΔnleE, Δnlec, ΔnleD |  | *This study* |
| SS7291 | ΔnleB, ΔnleE, Δnlec, ΔnleD, ΔespB | SS6108 deleted of espB | *This study* |
| SS7292 | ΔnleB, ΔnleE, Δnlec, ΔnleD, *espB-K179* | SS6108 with a 5-Codon insertion after K179 in espB. | *This study* |
| SS7293 | ΔnleB, ΔnleE, Δnlec, ΔnleD, *espB-E203* | SS6108 with a 5-Codon insertion after E203 in espB. | *This study* |
| SS7294 | ΔnleB, ΔnleE, Δnlec, ΔnleD, *espB-T239* | SS6108 with a 5-Codon insertion after T239 in espB. | *This study* |
| SS7295 | ΔnleB, ΔnleE, Δnlec, ΔnleD, *espB-L241* | SS6108 with a 5-Codon insertion after L241 in espB. | *This study* |
| SS7296 | ΔnleB, ΔnleE, Δnlec, ΔnleD, *espB-K282* | SS6108 with a 31-Codon insertion after K282 in espB. | *This study* |
| ***E. coli* K12 strains** |  |  |  |
| W3110 (237) | F^-^ λ^-^ rph-1 INV(*rrnD*, *rrnE*) |  | *Lab collection* |
| DY378 (1974) | W3110 λcI857, Δ(cro-*bioA*) | Expresses the lambda Red recombinase at 42°C. | *Ref. 50* |
| XTL634 (5768) | W3110 *araD*<>*tetA*-*sacB*-amp | Carrying the *tetA-sacB* cassette, used to amplify the cassette. | *Ref. 48* |

* All EPEC strains were derived from strain E2348/69, O127:H7.
