# Supplementary material for: Epithelial cells detect functional type III secretion system of enteropathogenic Escherichia coli through a novel NF-κB signaling pathway
Source: PLoS Pathog. 2017 Jul 3;13(7):e1006472. doi: 10.1371/journal.ppat.1006472 (PMC5510907; doi:10.1371/journal.ppat.1006472)
Supplement: S3 Table — (DOCX) [file ppat.1006472.s003.docx]

Table S3 - list of primers

| **Name** | **Sequence (5' to 3')** | **Usage** |
| --- | --- | --- |
| 1020 | ATGAATAAACTCTTAAATATATTTAAAAAAGCAGAGTCATATCACGATCTTGTGTAGGCTGGAGCTGCTTC | SK5174 |
| 1021 | TCATGCTCTGAAATCATTTACCGTTCCTAATACTTTAAGTTCGATATTATTCATATGAATATCCTCCTTAG | SK5174 |
| 1330 | CAAGTCATTAATACCAACAGCCTCTCGCTGATCACTCAAAATAATATCAACGTGTAGGCTGGAGCTGCTTC | SK4941 |
| 1331 | GGTACCTGGTTGGCTTTTGCCAGTACGGAGTTACCGGCCTGAACCTGCTGCCATATGAATATCCTCCTTAG | SK4941 |
| 697 | GATCAGAAGGACGTTGATCG | SK4973 |
| 680 | GTATTACCGCAAGGGTTTGG | SK4973 |
| 647 | CCGCAAGTTACTTCTCAACC | SK4986 |
| 692 | CCATCGTGAGGACTTTCATC | SK4986 |
| 721 | GTGCTCCTGACTCATAACAC | KB5004 |
| 1006 | GTATGGAGGCGTTACGAAGG | KB5004 |
| 685 | GCTGAAAGAGTACAACGCTG | SK5029 |
| 701 | GTACGACTGGATCCGTGGGTGTTTATGTTCTCCGCAGGATATCGTTTTTAAGTGTAGGCTGGAGCTGCTTC | SK5029 |
| 667 | CAACGCCGGATAATATGGAG | SK5075 |
| 681 | CTTGTGCCAGTGGTTATCTG | SK5075 |
| 670 | CTTTATGCAGTACTGCCTGC | SK5076 |
| 683 | CGAGATTTCAGCCAGTTGTC | SK5076 |
| 684 | GTGCGAAAAACCTGCTGATC | SK5077 |
| 700 | GATAGCGGTCATAAAACCGC | SK5077 |
| 686 | GGATGCCAAAGAACTGGTTG | SK5078 |
| 706 | GCAAGTGTCAGCATGGTATC | SK5078 |
| 673 | GTTCGTGATGTGACTGAAGAAG | SK5079 |
| 687 | CCATGTGGGTTTCGATGTTG | SK5079 |
| 676 | GTGAAGAGTCCCCAATAAGC | SK5080 |
| 688 | GTCTGTTCCGTCGTAAGAAC | SK5080 |
| 689 | GCATAGCAGGAATAGCAACC | SK5081 |
| 709 | CAGCACGATTATGAACGAGC | SK5081 |
| 690 | CCAGAACCTCAACGAAAACC | SK5082 |
| 712 | CATACACCACTTCCACCATC | SK5082 |
| 691 | GCGTAAGCGTTTGATGAAGC | SK5083 |
| 715 | GCTACGCAGCTTAGAGATTC | SK5083 |
| 694 | GCAACAAGGTGAAAGAGGAC | SK5084 |
| 718 | GTTTATGGTGGTCACGGTAC | SK5084 |
| 1498 | CAACGTTGCAGCATGGGTAACTCTTG | SK5430 |
| 1499 | CTGAATACGCCAGGGGTTGCAGGATGGG | SK5430 |
| 1005 | GCTCTTGCTTGAACCATTGC | SK5431 |
| 1006 | GTATGGAGGCGTTACGAAGG | SK5431 |
| 1007 | CGCTTCTCTGAATAGACGTG | SK5432 |
| 1008 | CGGTCATGTTGCTTTTGGTC | SK5432 |
| 942 | GGTGTATTTCCCGGCGATGG | SK5433 |
| 943 | CGAAGTTCATCCAGCATAGC | SK5433 |
| 1496 | GATGAGCAGTGTGGTAAAGGAGTGGC | SK5434 |
| 1497 | CAGGGGTAGCATCATCGAGCGG | SK5434 |
| 393 | GCATGGATCCTCATGATGTCATCCTGCGAACG | SK5566 |
| 544 | TCATTGTTGCAAGATGTCGC | SK5566 |
| 1824 | AGTCCTTCTGGCGGAGTATTACCACTGGCAGCCACAATCAATGGAAATGTTTCCTAATTTTTGTTGACACTCTATC | 6227 |
| 1698 | ATTTTATTTATGCGATGCGATGATTAGGCATATTTCATCGCTAATCCGCCATCAAAGGGAAAACTGTCCATA | 6227 |
| 1693 | GAGACAGGGGAATCTCTCATGATAAACGGACTTAATAATAATTCCGCATCCTAATTTTTGTTGACACTCTATC | 5966 |
| 1694 | GTCCATTTTTTTATTTTTAAATGTTTTTCAGGTATGTTTCAGATGCGCCATCAAAGGGAAAACTGTCCATA | 5966 |
| 1695 | TTAGATATAAAAAGGCATGAATTATGCTTAATGGAATTAGTCAAGCTGTTTCCTAATTTTTGTTGACACTCTATC | 5967 |
| 1696 | ATCTTTTAACTCTCTAAACAATCAACAGTAATTAATAATTTTAAATCTACATCAAAGGGAAAACTGTCCATA | 5967 |
| 1691 | GTTATATAGGGAGATGTATTATGTCGTCATCGTTATCAGGAATAATACTCTCCTAATTTTTGTTGACACTCTATC | 5968 |
| 1692 | TTATCTTCCGGCGTAATAATGTTTATTATCGCTTGAACTAATTTCCTCTATTTTCATCAAAGGGAAAACTGTCCATA | 5968 |
| 1699 | GAGTTCGCAGGATGACATCATGAATAAACTCTTAAATATATTTAAAAAAGCATCCTAATTTTTGTTGACACTCTATC | 5969 |
| 1700 | GTGGGTATCTTTCCAATACAGAATCATGCTCTAAAATCATTTACCGTTCCATCAAAGGGAAAACTGTCCATA | 5969 |
| 1925 | GAGACAGGGGAATCTCTCATGATAAACGGACTTAATAATAATTCCGCAGTGTAGGCTGGAGCTGCTTC | 6267 |
| 1926 | GTCCATTTTTTTATTTTTAAATGTTTTTCAGGTATGTTTCAGATGCGCCCATATGAATATCCTCCTTAG | 6267 |
| 1946 | TCGAGTTTAATTATTAAAGAGAATTTAATTATGAATACTATTGATTATACTAATCAAGTAGTGTAGGCTGGAGCTGCTTC | 6285 |
| 1947 | TTACCCAGCTAAGCGAACCGATTGACCCATACGATTCTGAAGATCAGTGAGATCGCGAGCCATATGAATATCCTCCTTAG | 6285 |
| 1852 | TAATCAGCCATACCACATTTG | 6196, 6197, 6198, 6199, 6200, 6201, 6202, 6203 |
| 1853 | TGATCTAGAGTCGCGGCC | 6196, 6197, 6198, 6199, 6200, 6201, 6202, 6203 |
| 1870 | GGCGGAGGGTCTGGGGGAGGCTCCATGGTGAGCAAGGGCGAGGAGG | 6205, 6206, 6207, 6208, 6209, 6210, 6211, 6212 |
| 1871 | GGTGGCGGGATCCCGGGC | 6205, 6206, 6207, 6208, 6209, 6210, 6211, 6212 |
| 1854 | CCGCGACTCTAGATCAATGAATTTATCTGAAATTACTCAAC | 6196 |
| 1855 | TGTGGTATGGCTGATTATTAAAAACTACGGTTAGAAATGG | 6196 |
| 1856 | CCGCGACTCTAGATCAATGGATGCATTATGCTATTG | 6197 |
| 1857 | TGTGGTATGGCTGATTATTATTGCATCGAAACTAATTTTG | 6197 |
| 1858 | CCGCGACTCTAGATCATTGACTAGAGTTTCTCTAAAAAGAAATTTG | 6198 |
| 1859 | TGTGGTATGGCTGATTATTAATTTTCATATTCAATTGTGAAC | 6198 |
| 1860 | CCGCGACTCTAGATCAATGGATACATCAACTACAGCATC | 6199 |
| 1861 | TGTGGTATGGCTGATTATTATTTACCAAGGGATATTCC | 6199 |
| 1862 | CCGCGACTCTAGATCAATGAATACTATCGATAATAACAATGC | 6200 |
| 1863 | TGTGGTATGGCTGATTATTACCCAGCTAAGCGAGC | 6200 |
| 1864 | CCGCGACTCTAGATCAATGCTTAATGTAAATAACGATATCC | 6201 |
| 1865 | TGTGGTATGGCTGATTATTAAACTCGACCGCTGAC | 6201 |
| 1866 | CCGCGACTCTAGATCAATGGAAGCAGCAAATTTAAG | 6202 |
| 1867 | TGTGGTATGGCTGATTATTAGGCATATTTCATCGC | 6202 |
| 1868 | CCGCGACTCTAGATCAATGAAAAAAATAATACTGAGTATCATTC | 6203 |
| 1869 | TGTGGTATGGCTGATTATTAATCGATAATTTGCTCATTATTC | 6203 |
| 1874 | CGGGATCCCGCCACCATGGAAGCAGCAAATTTAAG | 6205 |
| 1875 | GCCCTTGCTCACCATGGAGCCTCCCCCAGACCCTCCGCCGGCATATTTCATCGCTAATC | 6205 |
| 1876 | CGGGATCCCGCCACCTTGACTAGAGTTTCTCTAAAAAGAAATTTG | 6206 |
| 1877 | GCCCTTGCTCACCATGGAGCCTCCCCCAGACCCTCCGCCATTTTCATATTCAATTGTGAACTC | 6206 |
| 1878 | CGGGATCCCGCCACCATGGATGCATTATGCTATTG | 6207 |
| 1879 | GCCCTTGCTCACCATGGAGCCTCCCCCAGACCCTCCGCCTTGCATCGAAACTAATTTTG | 6207 |
| 1880 | CGGGATCCCGCCACCATGCTTAATGTAAATAACGATATCC | 6208 |
| 1881 | GCCCTTGCTCACCATGGAGCCTCCCCCAGACCCTCCGCCAACTCGACCGCTGACAATAC | 6208 |
| 1882 | CGGGATCCCGCCACCATGAAAAAAATAATACTGAGTATCATTC | 6209 |
| 1883 | GCCCTTGCTCACCATGGAGCCTCCCCCAGACCCTCCGCCATCGATAATTTGCTCATTATTC | 6209 |
| 1884 | CGGGATCCCGCCACCATGGATACATCAACTACAGCATC | 6210 |
| 1885 | GCCCTTGCTCACCATGGAGCCTCCCCCAGACCCTCCGCCTTTACCAAGGGATATTCCTG | 6210 |
| 1886 | GGGATCCCGCCACCATGAATTTATCTGAAATTACTCAAC | 6211 |
| 1887 | GCCCTTGCTCACCATGGAGCCTCCCCCAGACCCTCCGCCAAAACTACGGTTAGAAATGGTTG | 6211 |
| 1888 | CGGGATCCCGCCACCATGAATACTATCGATAATAACAATGCGGC | 6212 |
| 1889 | GCCCTTGCTCACCATGGAGCCTCCCCCAGACCCTCCGCCCCCAGCTAAGCGAGCCGC | 6212 |
| 1386 | CAAAAGTACTCGATGCCAATCATAAAGAACTGC | 5226 |
| 1387 | GCATGCGGCCGCTCATTTTTTGAGTGGGTGGATAT | 5226 |
| 1390 | CAAAAGTACTCGATGCCATCATTAGTTTCAGGTATTC | 5228 |
| 1391 | GCATGCGGCCGCTCACTTATCCTTTATGACAAAGTTTC | 5228 |
| 1392 | CAAAAGTACTCGATGTTACCAACAAGTGGTTCTTC | 5229 |
| 1393 | GCATGCGGCCGCTCATCCACATTGTAAAGATCCTTTG | 5229 |
| 1394 | CAAAAGTACTCGATGTTATCGCCCTCTTCTATAAA | 5230 |
| 1395 | GCATGCGGCCGCTTATATCTTACTTAATACTACACTAATAAGATC | 5230 |
| 1396 | CAAAAGTACTCGATGAACATTCAACCGATCGTAAC | 5231 |
| 1397 | GCATGCGGCCGCTTAGACTCTTGTTTCTTGGATTATATC | 5231 |
| 1400 | CAAAAGTACTCGATGATAAATGGCATTTCTCAAC | 5233 |
| 1401 | GCATGCGGCCGCTCAATTCCTCGAATATGCTTC | 5233 |
| 1404 | GCATGCGGCCGCATGCCTATTGGTAACCTTGG | 5235 |
| 1405 | GCATGCGGCCGCTTAAACGAAACGTACTGGTC | 5235 |
| 1406 | CAAAAGTACTCGATGTTTAGTCCAACGGCAATG | 5236 |
| 1407 | GCATGCGGCCGCCTACAGCCGAGTATCCTGCAC | 5236 |
| 1410 | CAAAAGTACTCGATGCTTAATGGAATTAGTAACGCT | 5238 |
| 1411 | GCATGCGGCCGCTTACCCTTTCTTCGATTGCTC | 5238 |
| 1872 | CGGGATCCCGCCACCATGATTAATGGGGTGTCG | 6204 |
| 1873 | GCCCTTGCTCACCATGGAGCCTCCCCCAGACCCTCCGCCGCAAAGCCTCTTACTCTTC | 6204 |
| 1232 | GATTTACATGCATTATGTGAGCTTCCGG | 4733 |
| 1233 | CCGGAAGCTCACATAATGCATGTAAATC | 4733 |
| 1899 | GGTGGCGGGATCCCGGGC | 6259, 6260 |
| 1900 | TACTGCGGCCGCGACTCTAGATC | 6259, 6260 |
| 1901 | CGGGATCCCGCCACCATGGTGAGCAAGGGCGAG | 6259, 6260 |
| 1902 | GTCGCGGCCGCAGTATTACTTGTACAGCTCGTCCATG | 6259 |
| 1903 | CCGTTCATCTTGTACAGCTCGTCCATGC | 6260 |
| 1904 | CTGTACAAGATGAACGGGGAGGCCATC | 6260 |
| 1905 | GTCGCGGCCGCAGTATTAACCAGGCTGCAGACG | 6260 |
| 3678 | ATGAATACTATCGATAATAACAATGCGGCAATCG | 7292, 7293, 7294, 7295, 7296 |
| 3679 | TTACCCAGCTAAGCGAGCCGCTT | 7292, 7293, 7294, 7295, 7296 |
| 3659 | TTACCCATACGATGTTCCAGATTACGCTTAGGTCGACCTGCAGCCAA | 7332 |
| 3660 | AAGCGTAATCTGGAACATCGTATGGGTAACTCTCCTTTTTCCGCCTCATGA | 7332 |
