# Supplementary figures and images for: Epithelial cells detect functional type III secretion system of enteropathogenic Escherichia coli through a novel NF-κB signaling pathway
Source: PLoS Pathog. 2017 Jul 3;13(7):e1006472. doi: 10.1371/journal.ppat.1006472 (PMC5510907; doi:10.1371/journal.ppat.1006472)

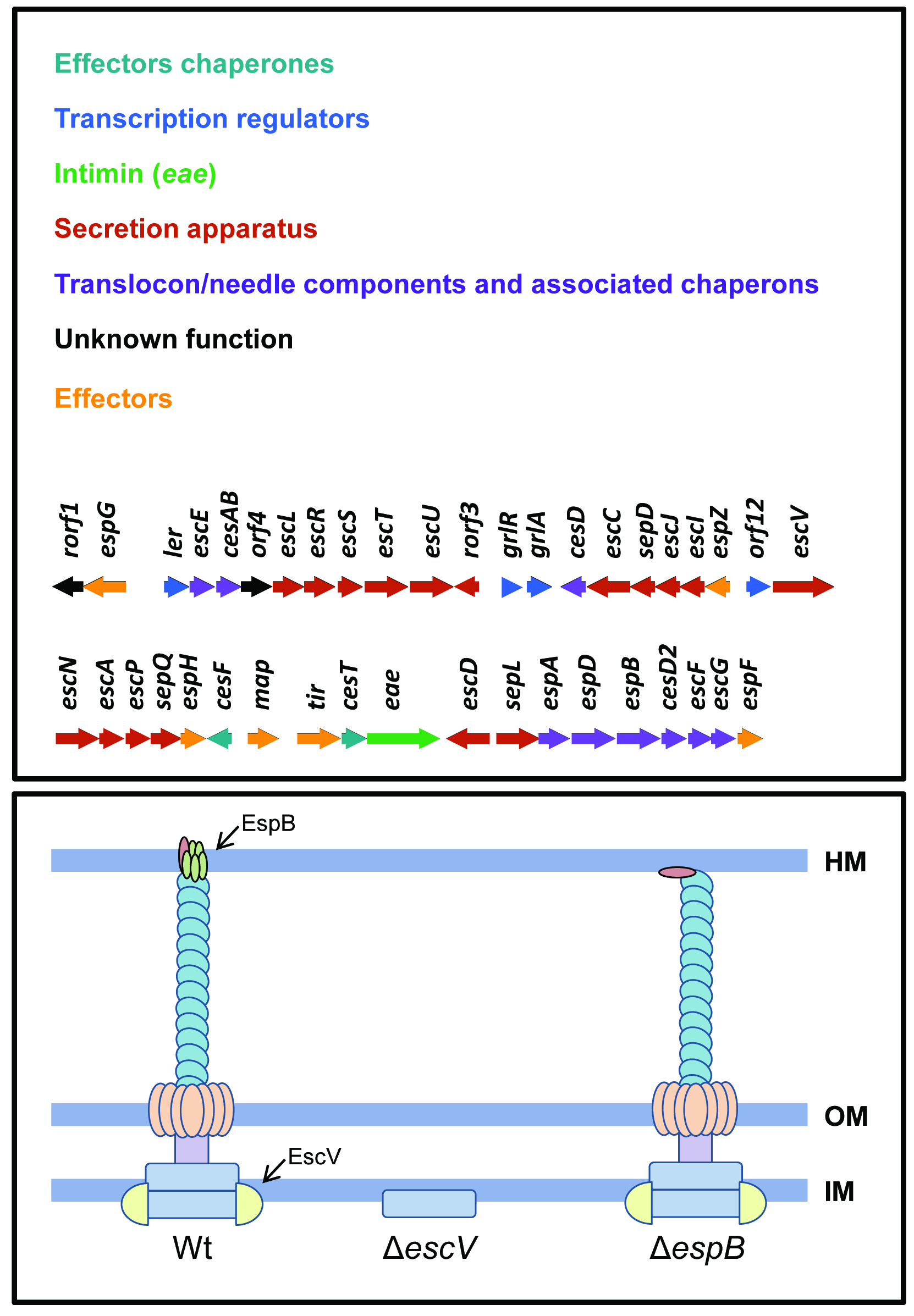

Supplement: S1 Fig — Upper panel: The locus of Enterocyte Effacement (LEE) is presented with color-coding depicting the nature of the genes encoded. Lower panel: Schematic diagram of the TTSS structures formed by wild type bacteria, the ΔescV mutant and the ΔespB mutant. Shown are the locations of the EspB and EscV proteins in the TTSS with respect to the bacterial inner membrane (IM), outer membrane (OM) and host cell membrane (HM). (TIF) [file ppat.1006472.s004.tif]

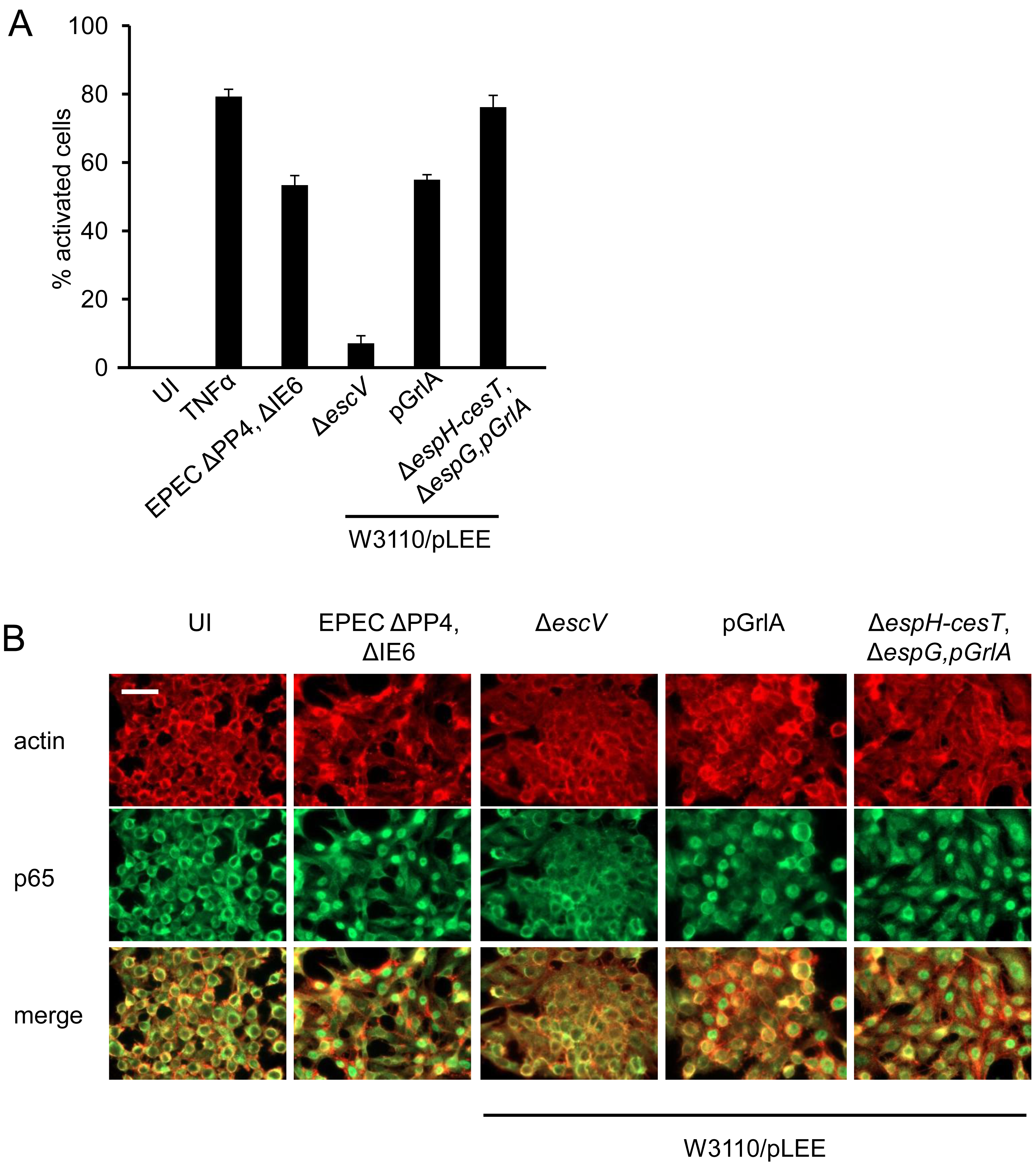

Supplement: S2 Fig — (A) T84 human colonic epithelial cells were infected with EPEC or with W3110 strains as indicated, or were treated with TNFα. The cells were then fixed, stained for p65 and quantified for nuclear p65 by microscopy. Representative images from this analysis (3 hours post infection) are shown in (B). Size-bar represents 200 microns. (TIF) [file ppat.1006472.s005.tif]

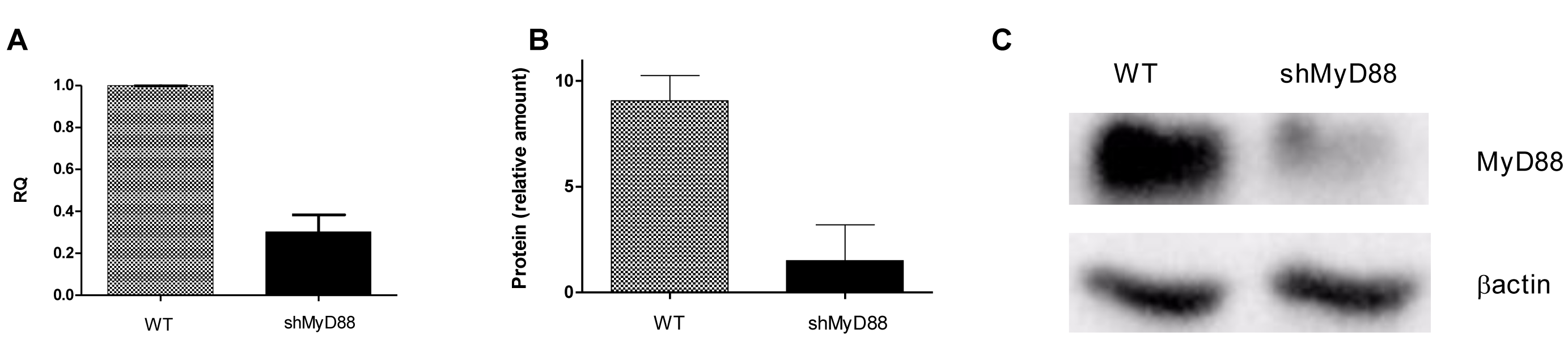

Supplement: S3 Fig — Wild-type (WT) and lentivirally transduced cells (shMyD88) were tested for MyD88 expression by qPCR and Western blot. (A) qPCR results normalized to beta-actin. (B-C) relative MyD88 protein by densitometry normalized for the amount of protein using beta actin as a control. (A) and (B) represent the average of three independent experiments. (C) One representative WB is shown. (TIF) [file ppat.1006472.s006.tif]

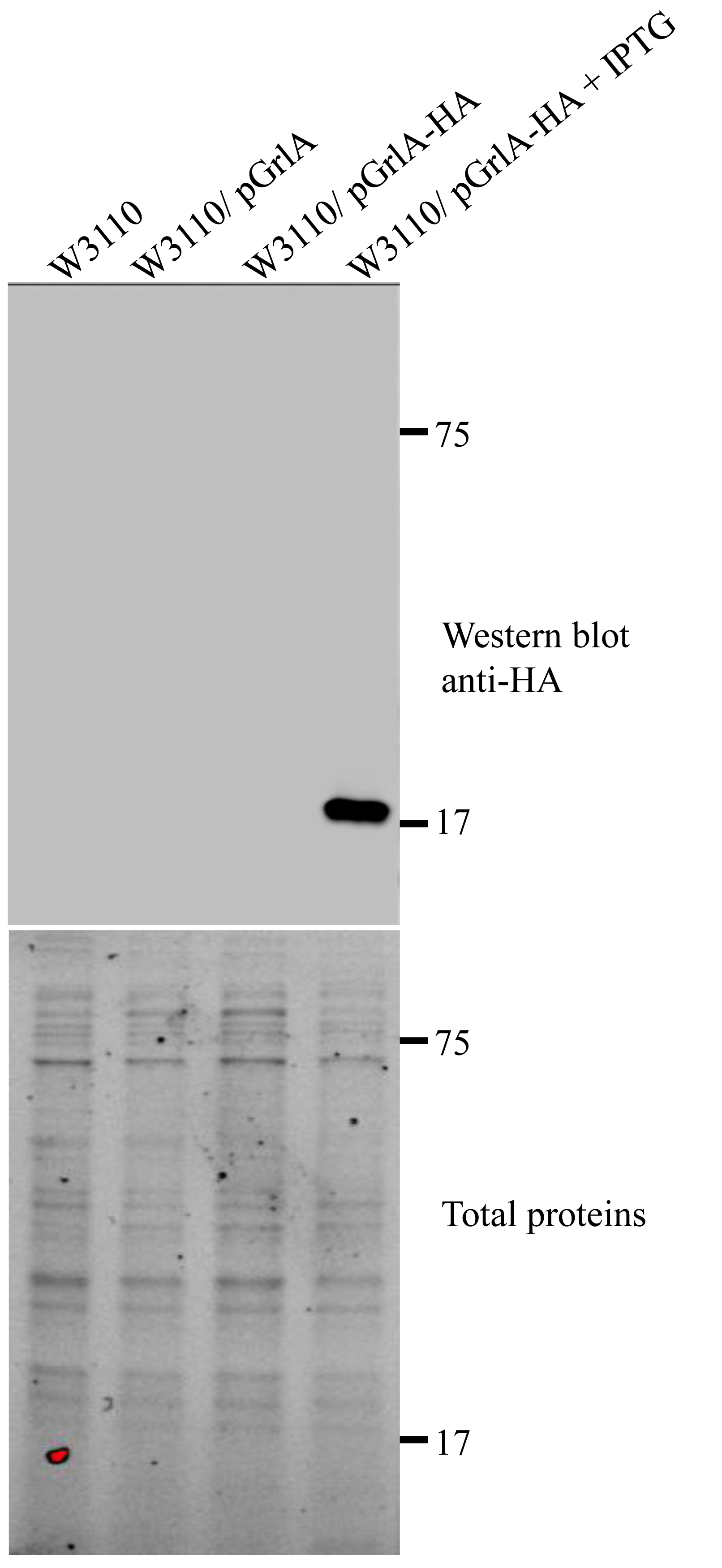

Supplement: S4 Fig — Bacteria containing, or not, plasmids expressing GrlA or GrlA-HA (as indicated), were treated, or not, with IPTG (as indicated). Proteins were extracted and resolved using Stain-FreeTM gel. After recording the amount of total proteins, the gel was used for Western blot analysis using anti HA antibody. The strains, plasmids and IPTG treatment are indicated above the lanes. Molecular size markers are shown on the right. (TIF) [file ppat.1006472.s007.tif]

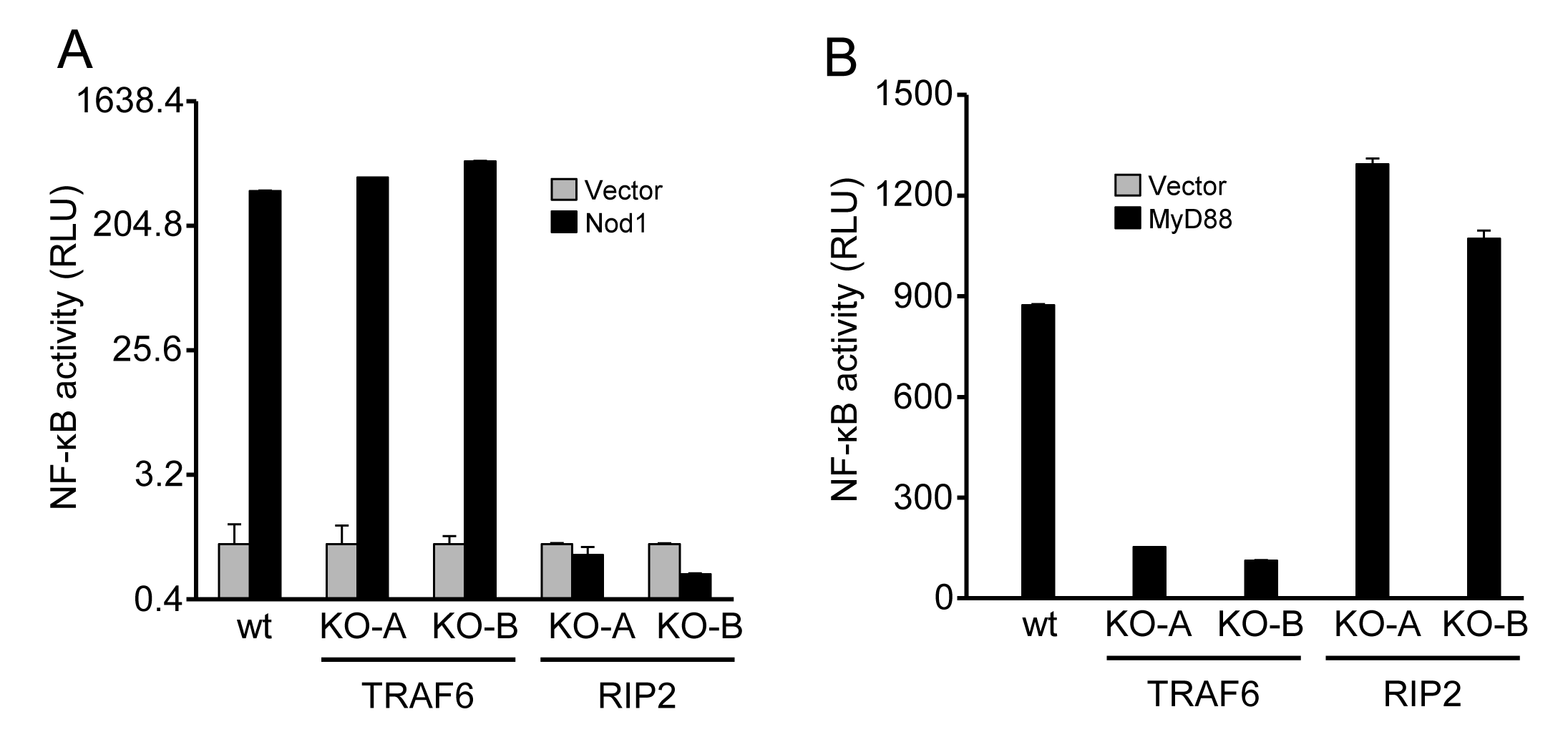

Supplement: S5 Fig — TRAF6-/- or RIP2-/- or wt HEK293 cells were co-transfected with the NF-κB reporter plasmid and a plasmid encoding Nod1 (A) or MyD88 (B), which activate NF-κB through the RIP2 and TRAF6 pathways, respectively, or an empty vector. NF-κB activation was determined by the dual luciferase assay. (TIF) [file ppat.1006472.s008.tif]
